# Drivers and determinants of strain dynamics following fecal microbiota transplantation

**DOI:** 10.1038/s41591-022-01913-0

**Published:** 2022-09-15

**Authors:** Thomas S. B. Schmidt, Simone S. Li, Oleksandr M. Maistrenko, Wasiu Akanni, Luis Pedro Coelho, Sibasish Dolai, Anthony Fullam, Anna M. Glazek, Rajna Hercog, Hilde Herrema, Ferris Jung, Stefanie Kandels, Askarbek Orakov, Roman Thielemann, Moritz von Stetten, Thea Van Rossum, Vladimir Benes, Thomas J. Borody, Willem M. de Vos, Cyriel Y. Ponsioen, Max Nieuwdorp, Peer Bork

**Affiliations:** 1 Structural & Computational Biology Unit, European Molecular Biology Laboratory, Heidelberg, Germany; 2 Centre for Digestive Diseases, Sydney, New South Wales Australia; 3 Genomic Core Facility, European Molecular Biology Laboratory, Heidelberg, Germany; 4 Department of Experimental Vascular Medicine, Amsterdam University Medical Centers, Amsterdam, the Netherlands; 5 Laboratory of Microbiology, Wageningen University, Wageningen, the Netherlands; 6 Human Microbiome Research Program, Faculty of Medicine, University of Helsinki, Helsinki, Finland; 7 Department of Gastroenterology & Hepatology, Amsterdam University Medical Centers, Amsterdam, the Netherlands; 8 Department of Vascular Medicine, Amsterdam University Medical Centers, Amsterdam, the Netherlands; 9 Max Delbrück Center for Molecular Medicine, Berlin, Germany; 10 Yonsei Frontier Lab, Yonsei University, Seoul, South Korea; 11 Department of Bioinformatics, Biocenter, University of Würzburg, Würzburg, Germany; 12 Present Address: The Novo Nordisk Foundation Center for Biosustainability, Technical University of Denmark, Kongens Lyngby, Denmark; 13 Present Address: The University of Queensland, School of Chemistry and Molecular Biosciences, St Lucia, Queensland Australia; 14 Present Address: Department of Marine Microbiology & Biogeochemistry, Royal Netherlands Institute for Sea Research, ‘t Horntje, the Netherlands; 15 Present Address: Key Laboratory of Computational Neuroscience and Brain-Inspired Intelligence (Fudan University), Ministry of Education, Shanghai, China

**Keywords:** Metagenomics, Clinical microbiology, Microbiome, Microbial ecology

## Abstract

Fecal microbiota transplantation (FMT) is a therapeutic intervention for inflammatory diseases of the gastrointestinal tract, but its clinical mode of action and subsequent microbiome dynamics remain poorly understood. Here we analyzed metagenomes from 316 FMTs, sampled pre and post intervention, for the treatment of ten different disease indications. We quantified strain-level dynamics of 1,089 microbial species, complemented by 47,548 newly constructed metagenome-assembled genomes. Donor strain colonization and recipient strain resilience were mostly independent of clinical outcomes, but accurately predictable using LASSO-regularized regression models that accounted for host, microbiome and procedural variables. Recipient factors and donor–recipient complementarity, encompassing entire microbial communities to individual strains, were the main determinants of strain population dynamics, providing insights into the underlying processes that shape the post-FMT gut microbiome. Applying an ecology-based framework to our findings indicated parameters that may inform the development of more effective, targeted microbiome therapies in the future, and suggested how patient stratification can be used to enhance donor microbiota colonization or the displacement of recipient microbes in clinical practice.

## Abstract (web summary)

Understanding the factors underlying colonization of donor microbes in recipients of fecal microbiota transplantation is a necessary first step to aid development of directed approaches that aim to couple colonization to clinical outcomes.

## Main

Fecal microbiota transplantation involves the transfer of gut microbes, viruses and luminal content to modulate a recipient’s microbiome, for therapeutic purposes. While the efficacy of FMT has been demonstrated for various diseases^1–3^, such as recurrent *Clostridioides difficile* infection (rCDI)^4,5^ or ulcerative colitis (UC^6,7^), it may also facilitate microbiome recovery following disturbance^8^ and can enhance microbiome-mediated responses to other therapies^9,10^. Nevertheless, despite demonstrable efficacy in a growing range of clinical applications, the mode of action of FMT remains poorly understood^3^ and neither clinical success nor adverse outcomes are currently predictable with accuracy.

Because FMT primarily targets the microbiome, the engraftment of ‘beneficial’ and/or displacement of ‘detrimental’ microbes are expected to cause clinical effects^3^, in conjunction with more specific processes of host–microbiome interplay, such as the modulation of immune responses^11^, restored short-chain fatty acid (SCFA) metabolism^12^ or reinstated phage pressure^13,14^. It has been argued that both microbiome engraftment and clinical success are mainly determined by donor factors, and that rationally selected ‘super-donors’ may improve therapeutic efficacy^15,16^. This donor-centric view has since been questioned, at least for some indications^17^, highlighting the importance of recipient^18–20^ or procedural^21^ factors instead.

Changes in microbial compositions following FMT have been studied with regard to phages^22^ or fungi^23,24^, yet the bulk of current knowledge is focused on bacteria and archaea where colonization by donor microbes and the persistence of indigenous recipient microbes emerge at the strain level of microbial populations^25^. Strain-level studies suggest that colonization levels following FMT vary across indications: whereas donor and recipient strains coexist long term in metabolic syndrome (MetS) patients^25^, donor takeover is the most common outcome in rCDI^26–28^, with intermediate outcomes in UC^29^ or obesity^30,31^. However, the factors shaping these differential strain-level outcomes remain poorly understood. In small pilot study cohorts, colonization success of donor strains leading to short-term persistence was associated with species phylogeny, broad microbial phenotypes and relative fecal abundances in rCDI^26,27^, but with more adaptive metabolic phenotypes in UC^32^.

Here we conducted a meta-analysis of novel and published metagenomes from fecal samples collected before and after FMT to compare the fate of donor and recipient strain populations across multiple disease indications. We hypothesized that drivers of FMT response are best studied from an ecological perspective:^33–35^ FMTs can be thought of as untargeted perturbation experiments on the gut microbiome in natura, pitting donor communities against those of the recipient, with outcomes that emerge from underlying ecological processes. We therefore quantified strain-level patterns of donor strain colonization, recipient strain resilience and turnover following FMT, both at the broad level of entire communities and specifically for individual species. We built cross-validated models to predict FMT outcome—defined here as colonization of donor strains and resilience of resident strains of the recipient—based on either ex ante variables (that is, knowable before the intervention) or post hoc readouts (measured after the intervention), further categorized by scope (procedural, donor related or recipient related) and resolution (host, community and strain level), yielding testable hypotheses. Linking informative variables and their predictive performance to putative underlying ecological processes, we provide a comprehensive view of host- and microbiome-level determinants of strain dynamics following FMT with relevance to gut microbial ecology in the clinical context and beyond.

## Results

### A meta-analysis of strain dynamics after FMT, across diseases

We analyzed a total of 1,492 fecal metagenomes collected in 316 time series of FMTs conducted for rCDI infection (*n* = 62 FMTs^26–28,32,36^), infection with extended-spectrum beta-lactamase-producing bacteria (ESBL, *n* = 59 (refs. ^37–39^)), MetS (*n* = 50 (refs. ^18,25,40^)), UC (*n* = 42 (refs. ^29,41–43^)), anti-PD1 therapy resistance in patients with melanoma (*n* = 37 (refs. ^9,10^)), irritable bowel syndrome (IBS, *n* = 30 (ref. ^44^)), Crohn’s disease (*n* = 18 (ref. ^45^)), chemotherapy-induced diarrhea in patients with renal carcinoma (*n* = 10 (ref. ^46^)), Tourette’s syndrome (*n* = 5 (ref. ^47^)) and in healthy volunteers (*n* = 3 (ref. ^48^)). Of these, 269 samples (from four independent cohorts) were metagenomically sequenced for this study (Supplementary Table 1).

Full sample triads (donor, recipient pre FMT as baseline and at least one post-FMT sample) were available for 228 of the 283 allogenic FMT cases in our study; the remaining 33 FMTs in the dataset were autologous transfers, of the recipient’s own stool; 3 ± 3 post-FMT samples were available per time series, with a final sampling time point on average 159.4 days after the intervention (Supplementary Tables 1–3 and Methods).

**Fig. 1:**
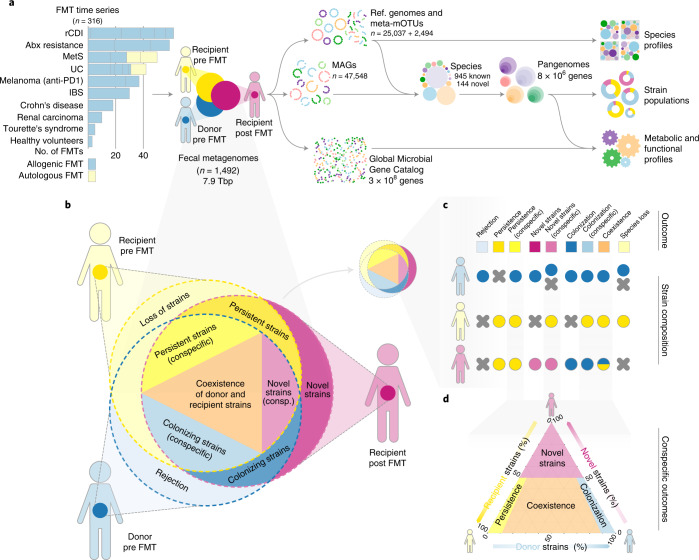
Study design and workflow overview. **a**, We analyzed a dataset of 316 FMT time series across ten disease indications and 22 cohorts, totaling 1,492 fecal metagenomes. Species pangenomes were built from reference genomes and newly generated MAGs and profiled across samples for taxonomic, functional and strain population composition, based on microbial SNVs and differential gene content. **b**, Each allogenic FMT was represented as a triad of donor pre-FMT (blue hues), recipient pre-FMT (yellow) and post-FMT (purple) samples; each sample’s strain population is indicated as an overlapping circle. **c**, FMT strain-level outcomes for each species were scored using patterns of determinant SNVs and gene content (Supplementary Table 5). **d**, Ternary diagram of the strain population space for conspecific recipient strain persistence, donor strain colonization, donor–recipient coexistence and influx of novel strains.

We profiled 1,089 microbial species, including 144 previously undescribed, via pangenomes (the total set of identified genes for a microbial species) constructed from 47,548 newly built metagenome-assembled genomes (MAGs) and 25,037 high-quality reference genomes (Fig. 1a and Methods). We compared the pre-FMT microbiome of recipients with their respective donors to identify single-nucleotide variants (determinant SNVs, as defined previously^25^) and differences in gene content, and used these (meta)genomic markers to evaluate the fate of donor and recipient strains in post-FMT samples (Fig. 1b,c). For each species we classified outcomes as: donor colonization (that is, the post-FMT strain population was dominated by donor strains); recipient persistence (dominated by recipient strains); coexistence of conspecific donor and recipient strains; influx of ‘novel’ strains not detected in baseline samples (representing the expansion of low-abundance strains, or introduction of new strains post FMT); donor rejection (failure to engraft at detectable concentrations); and loss of all recipient strains (Fig. 1c,d, Methods and Supplementary Table 5).

### Donor strain colonization is independent of clinical outcome

**Fig. 2:**
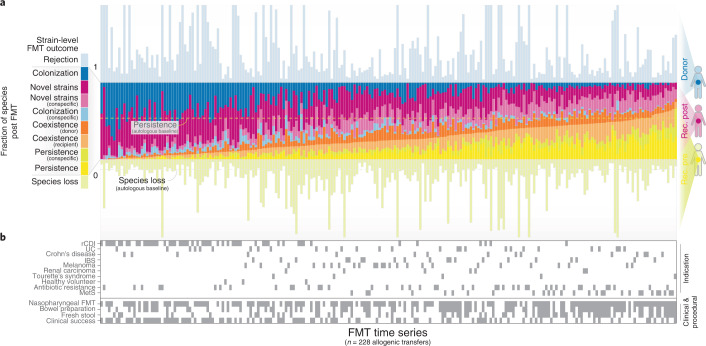
Community-wide FMT outcomes vary across patients and indications. **a**, Microbiome-level outcomes of 228 scorable allogenic FMT time series, summarized across all strain populations observed in donor and recipient (rec.). Fractions are normalized to the number of species observed in the recipient post FMT. **b**, Contextual data on indication, procedure and clinical outcome for each FMT time series in **a**.

Summarized across all tracked species, the colonization and persistence of donor and recipient strains, respectively, varied greatly among allogenic FMT patients (Fig. 2a,b). We observed neither complete recipient strain turnover (loss of all strains) nor complete donor rejection (failure to colonize) in any analyzed FMT instance, although persistence of recipient strains or colonization by donor strains was very low in some patients. Outcomes varied depending on the presence of the species before FMT: takeover by donor strains (accounting for 18.0 ± 16.0% species post FMT) and persistence of recipient strains (11.3 ± 9.1%) occurred more frequently among species present in either donor or recipient, but not in both. In contrast, in cases where species were present in both donor and recipient before FMT, coexistence of donor and recipient strains (19.0 ± 11.8%) was the most frequent outcome compared with donor colonization (4.5 ± 4.0%) and recipient persistence (5.6 ± 5.2%). Among post-FMT strain populations, 41.5 ± 21.0% were attributable to novel strains or entirely novel species not present in either donor or recipient pre FMT (or previously below detection limits). Such major turnover towards novel strains was probably associated with the intervention itself, because novel or previously undetected strains accounted for 50 ± 10.1% in autologous FMTs.

Takeover by donor and novel strains was characteristic of patients with rCDI or UC whereas MetS FMTs mostly resulted in conspecific strain coexistence, with varied outcomes in the other tested indications. Clinical response was not associated with strain-level dynamics for any indication; in other words, patient remission was not significantly linked to donor strain colonization or recipient strain displacement—for individual species and across all tracked species (Supplementary Fig. 1). In particular, our data did not support earlier hypotheses that reinstatement of SCFA production is a hallmark of remission in UC and rCDI, because an increased carriage of gut metabolic modules (GMMs; Methods) for acetogenesis, propionigenesis and butyrogenesis following FMT did not correlate with clinical outcome.

### Recipient, not donor, factors drive post-FMT strain dynamics

**Fig. 3:**
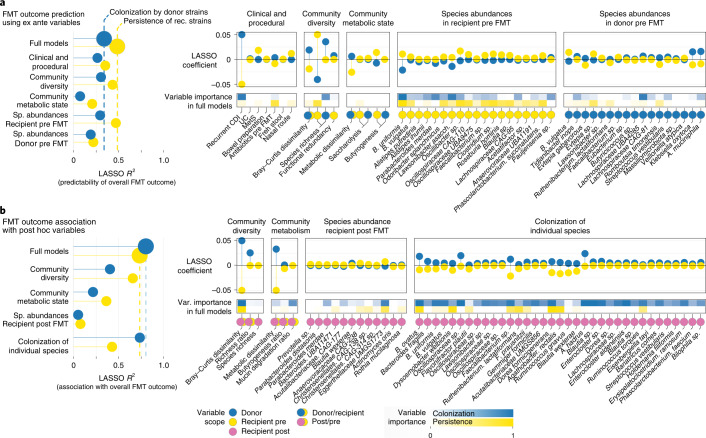
Drivers and determinants of FMT community-level outcomes. **a**, Ex ante predictability of microbial community-wide outcomes for individual FMTs (summarized across all trackable strain populations in a triad of donor, recipient pre-FMT and recipient post-FMT samples; Fig. 2) using cross-validated LASSO linear models with regularized subsets of different variable categories or a combination of all variables (‘full’ model) knowable before the intervention (Methods and Supplementary Table 6). Within each category, only the most relevant predictors are included. Predictive performance for each outcome index is shown as *R*^2^ on the left, and variable importance and directionality for the most predictive factors as cross-validated LASSO coefficients on the right. **b**, Association of FMT outcomes with LASSO-regularized sets of post hoc variables (measured after the intervention).

To identify factors associated with colonization outcome, we trained a series of predictive machine learning models using cross-validated LASSO-regularized linear regression (Methods). Among possible predictors we distinguished ex ante variables (that is, knowable before the FMT intervention; Fig. 3a) from post hoc variables (measurable after FMT; Fig. 3b). Moreover, we categorized predictors based on variable scope (procedural, donor related and recipient related) and resolution (host, community and species level), totaling >400 variables as regularization inputs (Supplementary Table 6). We then built cross-validated models for individual predictor categories (for example, using procedural variables only), as well as combined models to assess the overall predictability of outcomes.

Using regularized combinations of ex ante variables, the fractions of species exhibiting post-FMT coexistence of donor and recipient strains and post-FMT recipient strain persistence were predictable with moderate accuracy (LASSO *R*^2^ = 0.58 and 0.49, respectively), with lower variation explained for colonization by donor (*R*^2^ = 0.34) and pre-FMT recipient strain resilience (*R*^2^ = 0.35; Fig. 3a). Interestingly, the fraction of donor strains that successfully took over was not well predicted (*R*^2^ = 0.1309).

To identify the major determinants of strain outcomes, we compared the accuracy of models that used restricted subsets of variables with those of full models (which chose from all variables). Models that were restricted to community diversity indices (including species richness) or species abundances in the recipient before FMT achieved similar accuracies, reflecting the importance of these two factors in predicting the fate of donor and recipient strains after FMT. Moreover, across all models, variables capturing recipient factors or donor–recipient microbiome complementarity (for example, community dissimilarity) were more predictive than donor factors. The most important predictors of strain-level outcome included recipient species richness and abundances of selected species in the recipient before FMT, in particular *Bacteroides uniformis*, *Bacteroides vulgatus* and one *Oscillibacter* species, which were positively associated with overall recipient strain persistence and coexistence). In contrast, models based on procedural, metabolic or donor species variables were less accurate (Fig. 3a, left). Notably, donor carriage of GMMs related to SCFA synthesis was not associated with increased strain colonization, contrary to previous findings^12^. However, high carriage of butyrogenesis genes in the recipient before FMT was moderately associated with overall strain persistence—that is, recipient communities with higher butyrogenesis potential were generally more resilient, further highlighting the role of the recipient microbiome in post-FMT strain dynamics.

In the study population used here, rCDI state was associated with a higher fraction of successfully colonizing donor strains in the post-FMT microbiome. However, we note that while >90% of patients with rCDI in our dataset received antibiotics before intervention, most patients for other indications did not (or underwent extended washout periods), hence rCDI and the effect of antibiotics cannot be disentangled. Moreover, in full models choosing from all variables, higher species richness in the recipient and individual species abundances were more robust predictors for the persistence of recipient strains than rCDI state. This suggests that the high levels of donor strain colonization observed in patients with rCDI may be due in part to a more precarious microbial community (possibly instigated or exacerbated by antibiotic use), rather than being a disease-specific effect.

Models trained on post hoc variables were found to be highly accurate, in particular when describing donor colonization (Fig. 3b). As expected, the strength of community-wide compositional shifts in the recipient (Bray–Curtis dissimilarity and metabolic dissimilarity pre to post FMT) were associated with lower persistence of recipient strains. Interestingly, no individual species’ abundance post FMT was strongly associated with colonization outcome. However, successful colonization of particular species (Fig. 3b, right) was highly predictive of overall colonization of donor strains, in particular *B. uniformis*, *B. vulgatus*, several *Oscillospiraceae* sp. and *Lachnospiraceae* sp., including *Anaerostipes hadrus*. These might be considered indicator species, the successful engraftment of which is associated with an overall higher influx of donor strains.

### Post-FMT strain outcomes are species specific and predictable

**Fig. 4:**
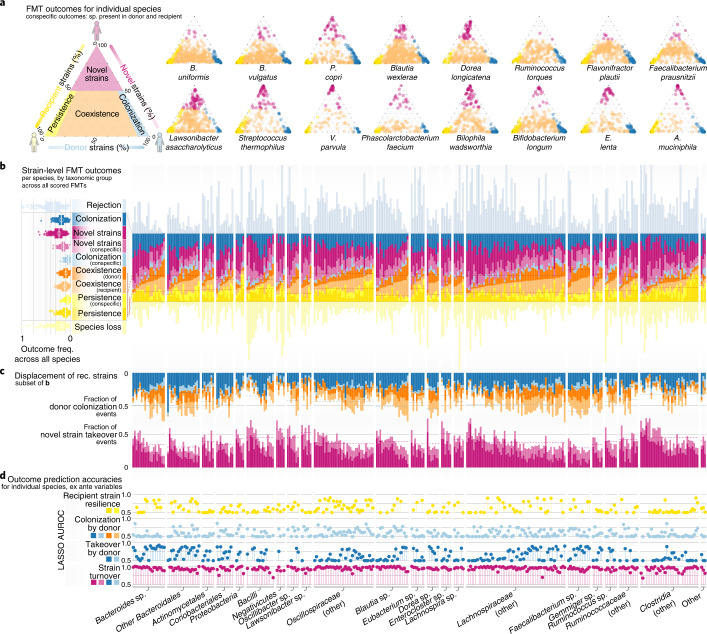
Strain-level FMT outcomes vary between species but are predictable ex ante. **a**, Strain-level outcomes for selected species are shown for conspecific FMT triads—that is, time series where the focal species was present in both donor and recipient pre FMT. Outcomes are scored as recipient strain persistence (dominance by recipient strains, yellow), donor takeover (blue), donor–recipient coexistence (orange) or influx of novel or previously undetected strains (purple), as indicated in the schematic on the left. Each dot corresponds to one scored FMT. **b**, Stacked bars representing outcomes for each species across scorable FMTs, scaled to the number of FMTs where the species was observed in the recipient following the intervention. Dashed lines indicate averages for recipient strain persistence within taxonomic groups (*x* axis). Outcome frequencies across all species are summarized on the left. **c**, Frequency of colonization by donor or novel (previously undetected) strains per species, as subsets of the data in **b**. Averages per taxonomic group are represented by dotted lines. **d**, Prediction accuracies of LASSO models for different binarized FMT outcomes (indicated on the left; Methods) as AUROC, averaged across cross-validation folds per species.

Whereas the above analyses describe summarized outcomes across all tracked species, we next investigated the strain population dynamics within each species post FMT. For sufficient statistical power, we focused on the 307 species detected in >50 allogenic FMTs across our study dataset (Fig. 4 and Supplementary Figs. 1 and 2). Recipient persistence, donor colonization, coexistence and influx of novel strains were observed for all species, with no notable phylogenetic signal. We did not observe any species with consistent patterns of colonization (‘super-colonizers’) or persistence (‘super-persisters’) across all FMTs. However, we observed two broadly distinct types of post-FMT strain dynamics in conspecific FMT triads (that is, for species present in both donor and recipient before the intervention; Fig. 4a and Supplementary Fig. 2). Most species showed a strong propensity towards donor–recipient strain coexistence that was independent of initial strain abundances. Notably, these included prevalent commensals like *Bacteroides* sp., *Blautia* sp., *Dorea* sp.*, Ruminoccocus* sp. and *Faecalibacterium* sp. In contrast, for *Veillonella parvula*, several *Streptococcus* spp., *Eggerthella lenta*, *Akkermansia muciniphila* and *Prevotella copri*, strain populations strongly tended towards dominance of either donor, recipient or novel strains, with infrequent coexistence, indicating that these species may be inherently less prone to conspecific strain carriage within the same host.

Strain-level FMT outcomes varied within each major taxonomic group, with no relevant differences between clades (Fig. 4b,c). Strains of facultatively aerobic species colonized less successfully (analysis of variance (ANOVA), *R*^2^ = 0.02, *P* = 0.002), whereas carriage of butyrogenesis (*R*^2^ = 0.026, *P* = 2 × 10^−4^) or propionigenesis (*R*^2^ = 0.008, *P* = 0.05) pathway genes or a generally saccharolytic (*R*^2^ = 0.046, *P* = 1.1 × 10^−6^) or proteolytic (*R*^2^ = 0.047, *P* = 8.5 × 10^−7^) metabolic setup was associated with higher colonization success.

To disentangle the factors contributing to post-FMT strain outcomes for each species, we built species-specific cross-validated logistic LASSO regression models using ex ante and post hoc sets of predictor variables, analogous to those discussed above (Fig. 4d). For each species we categorized strain-level outcomes, defining recipient resilience as events where recipient strains persisted (as dominant populations or coexisting with donor strains; yellow), donor colonization (donor strains successfully colonized as dominant or coexisting populations; light blue), donor takeover (donor strains become dominant; dark blue) and recipient turnover (dominance by donor strains and/or new or previously undetectable strains; purple). When training models using all available ex ante variables, recipient resilience (LASSO area under the curve (AUC) = 0.62 ± 0.13), donor colonization (0.58 ± 0.10) and donor takeover (0.65 ± 0.14) were predictable with moderate accuracy, with some variation within and between taxonomic clades (Fig. 4d). In contrast, recipient strain turnover (AUC = 0.94 ± 0.05) was predictable with high accuracy across almost all species, indicating that the displacement of resident strain populations in the recipient (not only by donor strain takeover, but by any means) may in general be a more deterministic process.

### Recipient microbiome drives species-specific strain dynamics

**Fig. 5:**
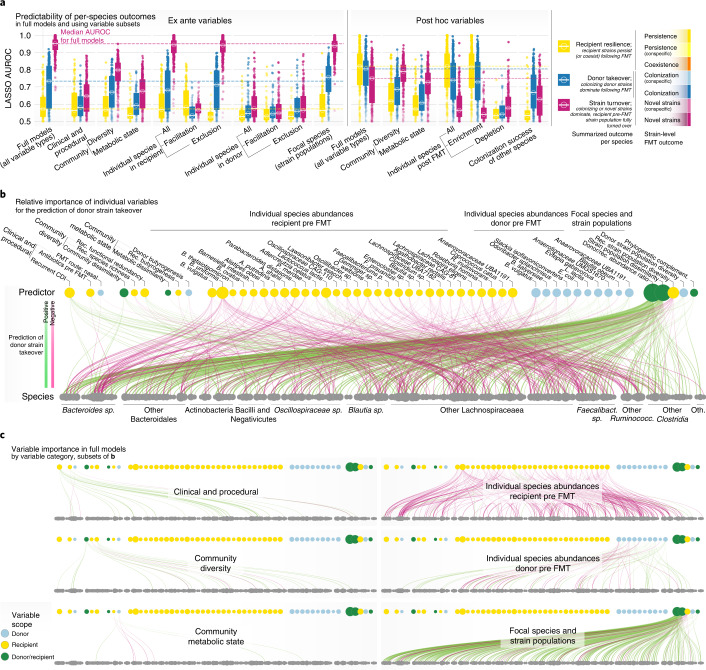
Drivers and determinants of FMT strain-level outcomes for individual species. **a**, Logistic LASSO models were trained to predict FMT binarized outcomes (recipient resilience, yellow; recipient turnover, purple; donor takeover, blue) for *n* = 307 species across FMT time series, using different subsets of ex ante variables (knowable before the intervention). Each dot represents data for one species. Data are shown for full models (choosing from all available variables) and models trained on variable subsets categorized by type (procedural, community-level diversity and so on). Predictive performance of species models is shown as average AUROC across LASSO cross-validation folds in marginal box plots, ranging from 0.5 to 1.0; center line, median; box limits, upper and lower quartiles; whiskers, maxima/minima within 1.5× interquartile range from upper/lower quartiles. **b**, Variable importance across full models to predict takeover by donor strains. Each edge indicates the importance of a predictor variable (top row) when predicting donor takeover for a given species (bottom row). Dot size for predictors indicates summed variable importance across all species; dot size for species (bottom) indicates total number of relevant predictors. Edge color and width indicate direction and strength of the association, respectively. **c**, Variable importance for individual predictor categories, as subsets of the data in **b**.

We built LASSO models that were restricted to different subcategories of predictor variables and compared their performance with full models trained on the entire complements of ex ante or post hoc variables (Fig. 5a). Models trained exclusively on recipient pre-FMT species abundances, on abundance and strain population characteristics of the focal species and, to a lesser degree, on microbiome community diversity variables achieved highest accuracies, comparable to those of full models. Notably, predictive power of individual recipient species was due almost entirely to exclusion effects, meaning that the enrichment of certain species in the recipient was associated with less donor takeover or recipient strain turnover of others, while facilitation effects did not have a contributing role. Models restricted to procedural factors (including disease indication), pre-FMT metabolic state or donor species abundances achieved much lower accuracies than full models, indicating that these variable groups were less predictive of strain-level outcomes. Overall, we observed similar trends for models trained on post hoc variables (Fig. 5a, right).

For most species, we found that strain turnover could be accurately predicted using only two community-level microbiome diversity measures—species richness in the pre-FMT recipient and donor–recipient community dissimilarity, the main factors selected in models restricted to community diversity variables (Fig. 5b). Low richness and a strong compositional shift in the recipient microbiome relative to healthy donors are hallmarks of disease-associated microbiome states, and our data indicate that the strength of this diffuse imbalance, correlated to disease (such as rCDI or UC in our dataset) or other disturbances (for example, antibiotics pretreatment or bowel cleansing), is directly linked with FMT outcome in most species. In contrast, donor richness or functional redundancy, previously proposed to be relevant^49^, were only subordinately predictive, if at all. Metabolic variables were likewise unreliable predictors. Community-wide butyrogenesis potential was negatively associated with turnover in the recipient (that is, strain populations were more resilient in recipients carrying high loads of butyrate production genes), but higher butyrogenesis levels in the donor did not correspondingly promote colonization. However, in full models for recipient strain turnover, these variables were superseded by indicator species in the recipient microbiome (see below) and focal species characteristics (in particular, recipient strain population diversity; Fig. 5b).

The strongest predictor of takeover by donor strains was a high donor/recipient abundance ratio of a species (as suggested previously for rCDI^27^), indicating that the amount of incoming viable donor microbes (also referred to as propagule pressure) may provide a neutral baseline estimate for donor strain colonization success, in particular for species not present in the recipient pre FMT (Fig. 5b,c). In general, while the donor/recipient ratio was most predictive, the underlying signal was driven by species abundance (or absence) in the recipient microbiota, much less so in the donor microbiota. Intraspecific strain population properties—donor/recipient strain population dissimilarity and recipient (and, to a much lesser extent, donor) strain population diversity—were also highly predictive but effects were more nuanced: donor strain takeover was more likely in species with complementary strain populations between donor and recipient, while diverse recipient populations (not dominated by individual strains) were more resilient than uneven ones. Moreover, incoming species that were phylogenetically complementary to the recipient community (that is, adding novelty—for example, by filling an unoccupied niche) were more likely to colonize or turn over the resident population.

### Resident ‘gatekeeper’ species inhibit donor strain engraftment

Given that FMTs involve the pitting of the recipient’s residual microbial community against incoming microbiota from the donor, we specifically explored the impact of individual species on the engraftment of others by training models restricted to donor or recipient pre-FMT species abundances (Fig. 5a) and exploration of individual species’ relevance as predictors in full models (Fig. 5b,c). We extracted networks of engraftment inhibition and facilitation, associating the abundance of putative effector species in the donor and recipient with donor takeover events in focal species. The vast majority of interactions was inhibitive (Fig. 5a–c): for most species, higher abundance in both donor and recipient correlated negatively with engraftment of other species. These exclusion effects were stronger for the resident community of the recipient (AUC = 0.63 ± 0.14) than the donor (AUC = 0.53 ± 0.06).

Colonization inhibition was phylogenetically concentrated—that is, inhibitive interactions were more common between related species within the same clade than between clades (Fig. 5B). Bacteroidales in the recipient microbiota, in particular *B. uniformis*, *B. vulgatus, Alistipes shahii* and *Parabacteroides distasonis*, were among the strongest colonization inhibitors, but also included two of the most strongly inhibited species, *Bacteroides xylanisolvens* and *Bacteroides ovatus*. In other words, the enrichment of gatekeeper species such as Bacteroidales in the recipient microbiota inhibited colonization for a broad panel of species, and vice versa, in line with previous findings that subgroups of Bacteroidales are generally highly persistent also in healthy individuals^50^. *Lactococcus lactis, Streptococcus salivarius* and *Dialister invisus* in the recipient were the foremost colonization facilitators. In contrast to colonization inhibition, facilitation typically affected phylogenetically distant species—for example, the facilitation of *Paraprevotella clara* and *Erysipelatoclostridium ramosum* colonization by recipient *Pauljensenia* sp. (an Actinobacterium) were among the strongest interactions observed across all species.

We observed few prominent predictive species in the donor microbiota, most notably *B. vulgatus* and *Evtepia gabavorous*. Facilitation and inhibition effects of donor species were generally limited and overall less predictive of colonization success, indicating that the donor microbiota has limited impact on colonization outcome beyond intraspecific strain dynamics.

### Adaptive and neutral processes shape the post-FMT microbiome

**Fig. 6:**
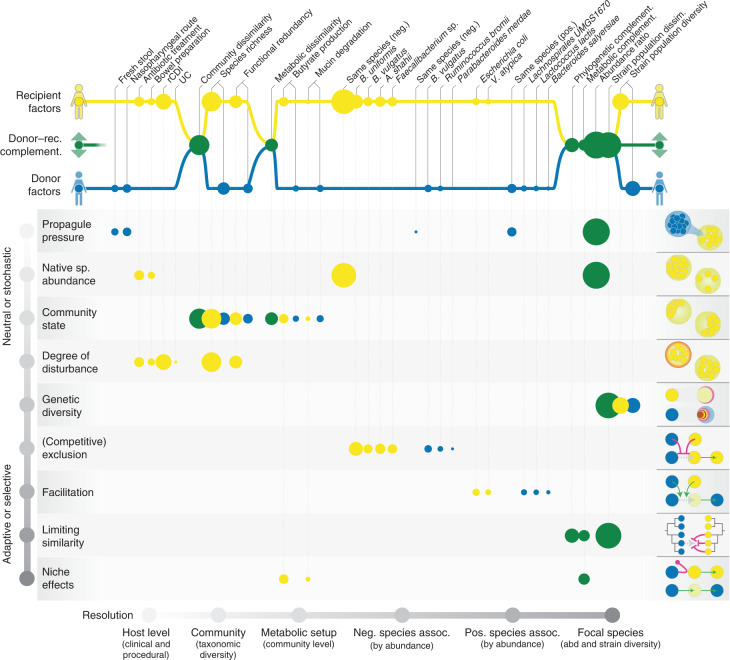
FMT strain-level outcomes are shaped by both neutral and adaptive processes. Each of the tested variables used to predict FMT outcome can be linked to putative underlying ecological processes, as suggested previously^33^. Factors are organized by scope (pertaining to the donor, recipient or donor–recipient complementarity, top) and resolution (host, community, species and strain level; left to right). Underlying ecological processes can be roughly ranked along the gradient, from neutral/stochastic to adaptive/selective; each process is illustrated with a toy example on the right. Circle size corresponds to average variable importance, calculated across all tested species from LASSO coefficients and overall model performance (less predictive models penalize variable importance). Recipient factors and, in particular, donor–recipient complementarity measures across all resolutions, were generally far more relevant to species-level outcome than donor factors. neg, negative; pos., positive; abd, abundance.

The accurate prediction of strain-level outcomes after FMT is informative beyond mere descriptive associations when construed through the lens of gut ecology: FMTs are community-level perturbation experiments, interpretable in a framework of invasion ecology and community assembly to identify processes and mechanisms that shape the microbiome^33–35^. We therefore linked the various tested variables in our models to putative underlying mechanisms (Fig. 6), categorized along a gradient from neutral/stochastic factors (for example, donor propagule pressure: the amount of incoming viable donor microbes) to adaptive/selective ones (for example, niche effects). We further distinguished recipient-specific, donor-specific and donor–recipient complementarity effects and organized variables by granularity, from host-level factors (for example, clinical or procedural) to the level of microbiome communities (overall composition and possible species interactions) and intraspecific (strain-level) effects.

Factors pertaining to the recipient or to donor–recipient complementarity were far more relevant to FMT colonization outcome than donor readouts across all tested variables, and consistently across different species. In other words, as outlined above, the donor microbiome did not specifically influence colonization or turnover in its own right, but instead mattered only to the extent of its complementarity with the recipient microbiota. Donor/recipient abundance ratios were highly determinant of FMT outcome, interpretable as the balance between propagule pressure of incoming donor cells and native abundance of the residual recipient population, providing a baseline of how neutral mechanisms shape post-FMT communities. In this, exclusion effects by resident strains of the recipient were dominant—that is, depletion of the recipient’s microbiota is more relevant for successful colonization than a higher dosage of donor strains. In practice, this interplay may be modulated procedurally to some extent, for example, by the use of fresh versus frozen stool (impacting the viability of donor cells), FMT route (rectal or duodenal) or the purging of recipient communities via bowel preparations or antibiotic pretreatment, although these procedural variables were not in themselves robust predictors in our analysis, possibly because they were confounded with the individual studies included in the dataset.

Microbiome composition of recipients (but not their respective donors) was likewise relevant to FMT strain-level outcome: broad community depletion (low richness) and pronounced compositional differences in regard to healthy donors may indicate generally disturbed and precarious microbiomes that are less resistant to takeover by donor strains. Conversely, the residual enrichment of gatekeeper species, such as *B. uniformis* or *B. vulgatus*, was also negatively associated with colonization by donor strains, possibly indicating competitive exclusion processes and interspecific priority effects. While by design, causality cannot be inferred from our data, these results tie in with existing ecological theories on microbiome stability and resilience—for example, on tipping elements and critical transitions^51,52^, community multistability leading to enterotypes^53,54^, priority^55^ or ‘Anna Karenina’ effects^56^. We found limited evidence for colonization facilitation across species boundaries, both in donor and recipient. Likewise, our data did not support a strong role for community-wide metabolic states: neither general metabolic setup nor specific metabolic modules such as SCFA production in donor or recipient greatly impacted FMT outcomes.

The strongest effects toward donor strain colonization emerged at species and strain level. Incoming species were more likely to colonize if they were phylogenetically or metabolically complementary to the residual community, implying that they were able to take over unoccupied niches. Colonization success was associated with complementarity specifically to the local community. High conspecific diversity in the donor and low diversity in the recipient were also linked with engraftment success: recipient populations dominated by single strains were less resilient, and donor strains from more diverse panels were more likely to colonize, probably due to strain-level-limiting similarity effects. Indeed, conspecific donor strain populations colonized more successfully if they were dissimilar to recipient strains, indicating strong inhibitive intraspecific priority effects.

However, we note once more that the colonization of individual species was predictable with only moderate accuracy, irrespective of the variable sets used—unlike residual strain population turnover, which was highly predictable. This implies that colonization success may be stochastic to a large extent.

## Discussion

Fecal microbiota transplantations are clinical procedures that can also be thought of as complex in natura perturbation experiments, pitting gut microbial communities of the donor against those of the recipient. An FMT is considered to be clinically successful if it triggers patient remission or recovery, whereas success from an ecological perspective is the extent to which the donor’s microbiota can colonize in the recipient. Given that FMT targets the gut microbiome, engraftment and clinical success are expected to correlate, implying that successful microbiome modulation mediates clinical effects. However, this hypothesis had not previously been systematically tested and is indeed not supported by our data. In our meta-study of 316 FMTs, clinical success was associated neither with colonization by donor strains, displacement of recipient species nor the reinstatement of specific functions (such as SCFA synthesis) for any of the studied disease indications. To some extent, this is in line with previous observations that autologous FMTs^57,58^ or even transfers of sterile-filtered fecal water^59^ can be efficacious. Our data do not rule out more subtle links, in particular given our limited sample size per indication and differences between FMT protocols across studies, but a clear role of donor microbiota colonization in shaping clinical responses did not emerge. We did observe overall higher levels of donor strain colonization in patients suffering from rCDI or UC, coinciding with higher clinical response rates in these diseases compared with others in our dataset. However, this was arguably due to overall more perturbed microbiome states associated with these diseases (possibly instigated by antibiotic treatment regimes) that outweighed disease-specific effects: we found no significant differences in strain-level outcomes between clinical responders and nonresponders to FMT.

Understanding microbiome-level FMT outcomes is both clinically relevant (for example, for informed donor selection or to avoid possible adverse effects) and more generally informative of ecological processes shaping the gut microbiome. All studied species exhibited all FMT outcomes, depending on context; we did not find strong evidence that any species was inherently more invasive or resilient than others. Rather, fine-scale intraspecific strain population structure and diversity, as well as donor–recipient strain population complementarity, determined resilience, coexistence and colonization, although we noted that while the majority of species tended towards conspecific donor–recipient strain coexistence, a smaller subset of species generally gravitated towards dominance by either recipient or donor strains or those undetectable at baseline. Interactions between species were less relevant, but clearly structured: several gatekeeper species in the recipient, in particular of the genus *Bacteroides*, inhibited colonization by other, phylogenetically unrelated species whereas colonization facilitation across species boundaries was scarce.

We found that the turnover of recipient strains was very accurately predictable for almost all studied species, using a consistent and surprisingly small selection of ex ante microbiome variables. In contrast, our models achieved only moderate predictive accuracies when predicting takeover by donor strains, indicating that colonization is, to a large extent, stochastic or influenced by other factors outside the scope of our study, such as viral or eukaryotic microbiome members, recipient immune state, medication or reduced viability of anaerobic donor fecal cells following the intervention.

Recipient factors consistently outweighed donor factors in driving FMT strain-level outcomes. Thus, our data did not support the super-donor hypothesis^15^ which states that certain donor microbiome properties are crucial to colonization and, by proxy, clinical success. Rather, we found that complementarity of donor and recipient microbiomes promoted donor colonization and recipient turnover. This phenomenon was observed across microbial resolutions, from community-level effects to conspecific strain population dissimilarity. Indeed, strain-level diversity and complementarity were the strongest determinants of FMT outcome, with relevance to rational donor selection in clinical practice^16,35^. Beyond screening for donor health, matching of donors to recipients based on microbiome complementarity at community, species and, in particular, strain levels may increase colonization success, make clinical outcomes more predictable and reduce adverse effects.

Our data suggest that the gut microbiome is shaped by both neutral and adaptive processes post FMT, reconciling previous reports^27,32^. We found that limits to gut microbiome resilience at community, species and strain level can be defined by a relatively small set of measurable variables that point to distinct underlying processes. The (complementary) interplay between propagule pressure and residual species abundance provided a neutral baseline for colonization although, again, recipient effects outweighed donor effects. At the same time, our data also suggested niche effects, in particular at the level of complementary intraspecific strain populations, although no consistently adaptive traits emerged in the analysis. Previous hypotheses pertaining to the importance of metabolic capabilities such as SCFA synthesis were not supported, although we note that the inference of SCFA biosynthesis pathways from metagenomic data remains challenging and does not capture putatively differential expression of SCFA synthesis genes.

By design, our study is predominantly descriptive and only probes potential mechanisms underlying our observations to the extent of interpreting them in an ecological context. Moreover, our study focuses on the bacterial and archaeal microbiota (not accounting for viruses and eukaryotes^60^) and is limited by the relatively small available sample size for some disease indications in our dataset, by the technical, procedural and physiological heterogeneity between cohorts and by the inherent detection limits of metagenomic strain calls. Nevertheless, our core findings were robust in spite of these sources of variation and may thus inform the clinical use of FMT in several ways, in particular if microbiome modulation is a desired endpoint beyond alleviation or remission of symptoms. Patients may be stratified before the intervention based on surprisingly crude, robust and easily obtainable microbiome readouts, such as community richness and high-level composition, or with regard to the presence of gatekeeper species associated with overall microbiome resilience. The relevance of donor selection, in contrast, appears mostly limited to the extent of the donor’s (strain-level) complementarity to the recipient. Tuning of procedural parameters (antibiotic pretreatment, stool preparation, dosage, FMT route, dietary intake of donors and so on) may mainly impact recipient microbiome resilience, and an overall more resilient response (excluding, of course, target pathogens to be displaced) is often desirable. Both inhibition and facilitation of colonization across species boundaries were surprisingly sparse and mild, with few exceptions, indicating that the targeted colonization or turnover of individual species may be achievable mostly independent of residual and cotransferred communities, minimizing collateral effects on the recipient’s microbiota.

Our results indicate that microbiome dynamics following FMT are impacted by defined parameters that are tunable in clinical practice, thus supporting the notion that predictable and efficacious microbiome modulation using personalized probiotic mixtures, rather than entire complex fecal samples, is possible and may profit from an ecological perspective. In particular, our findings suggest that the targeted depletion of selected microbes in the recipient, with concurrent introduction of diverse strain populations of the same species rather than a single strain, presents a promising approach to enhancing colonization and turnover in the recipient, although links to clinical outcomes remain to be established. Thus, levering of both neutral and relevant adaptive ecological processes may pave the way towards targeted modulatory interventions on the gut microbiome, personalized to patients, with predictable microbiome-level outcomes.

## Methods

### Data overview

The study dataset comprised 22 independent cohorts recruited in centers in the United States, the Netherlands and Australia, with a total of 316 FMTs conducted in 311 patients suffering from rCDI (*n* = 62 FMTs^26–28,32,36^), infection with ESBL (*n* = 59 (refs. ^37–39^)), MetS (*n* = 50 (refs. ^18,25,40^)), UC (*n* = 42 (refs. ^29,41–43^)), anti-PD1 therapy resistance in patients with melanoma (*n* = 37 (refs. ^9,10^)), IBS (*n* = 30 (ref. ^44^)), Crohn’s disease (*n* = 18 (ref. ^45^)), chemotherapy-induced diarrhea in patients with renal carcinoma (*n* = 10 (ref. ^46^)), Tourette’s syndrome (*n* = 5 (ref. ^47^) and in healthy volunteers (*n* = 3 (ref. ^48^)). On average, 4.11 recipient stool samples were available per FMT time series, including baseline samples taken before the intervention (pre-FMT). Overall, 7.9 Terabases (Tb) of sequencing data were analyzed across 1,492 fecal metagenomes, of which 269 (for 76 time series) were generated as part of the present study (for cohorts UC_NL, ESBL_NL, MetS_NL_1 and div_AU).

Three cohorts (UC_NL, MetS_NL_1 and MetS_NL_Koopen) were randomized controlled trials during which a subset of patients received autologous FMTs (transplantation of the recipient’s own stool, *n* = 33 FMTs). All other FMTs (*n* = 283) were allogenic, using stool donors. For 228 FMT time series, a full complement of donor baseline, recipient baseline and at least one recipient post-FMT sample were available after filtering.

A full description of all cohorts is provided in Supplementary Table 1, detailed information per FMT time series in Supplementary Table 2 and per-sample information in Supplementary Table 3.

### Sample collection, processing and metagenomic sequencing

Study design and fecal sample collection for cohorts MetS_NL_1 (refs. ^18,25^), UC_NL^41,61^ and ESBL_NL^37^ were described previously. rCDI_AU and UC_AU samples were obtained from a single-center, proof-of-concept, parallel and controlled study in collaboration with the Centre for Digestive Diseases (Sydney, Australia), which aimed to assess donor microbiota implantation in two patients with CDI and three with UC up to 28 days following a 2-day fecal microbiota transplantation infusion via transcolonoscopy and rectal enema. The study is registered with the Australian New Zealand Clinical Trials Registry under ACTRN12614000503628 (Universal Trial no, U1111-1156-5909). Written, informed participant consent and ethical approval were obtained via the Centre for Digestive Diseases Human Research Ethics Committee. Deidentified participant data relevant to the study are provided in Supplementary Tables 2 and 3.

For cohorts MetS_NL_1 and UC_NL, fecal DNA extraction was described in the original studies. DNA from ESBL_NL samples was extracted using the GNOME DNA Isolation Kit (MP Biomedicals) with the following minor modifications: cell lysis/denaturation was performed (30 min, 55 °C) before protease digestion was carried out overnight (55 °C), and RNAse digestion (50 μl, 30 min, 55 °C) was performed after mechanical lysis. After final precipitation, DNA was resuspended in TE buffer and stored at −20 °C for further analysis.

Metagenomic sequencing libraries for MetS_NL_1, UC_NL, ESBL_NL and div_AU samples were prepared to a target insert size of 350–400 base pairs (bp) on a Biomek FXp Dual Hybrid with high-density layout adapters, orbital shaker, static peltier and shaking peltier (Beckman Coulter) and a robotic PCR cycler (Biometra), using SPRIworks HT kits (Beckman Coulter) according to the supplier’s recommendation, with the following modifications: 500 ng of DNA initially, adapter dilution 1:25, kit chemical dilution 1:1 in process. For samples with low-input DNA concentrations, libraries were instead prepared manually using NEBNext Ultra II DNA Library Prep kits with NEBNext Singleplex primers. Libraries were sequenced on an Illumina HiSeq 4000 platform with 2 × 150-bp paired-end reads.

### Public datasets

Based on a literature search, 18 datasets on FMT cohorts that met the following criteria were included in the study: (1) public availability of metagenomic sequencing data in January 2022; (2) sufficient available description to unambiguously match donors and recipients per FMT time series; and (3) no restrictions on data reuse. They were included in this study as RCDI_US_Smillie (*n* = 22 FMT time series^26^), RCDI_US_Aggarwala (*n* = 14 (ref. ^28^)), RCDI_US_Watson (*n* = 10 (ref. ^32^)), RCDI_US_Podlesny (*n* = 8 (ref. ^27^)), RCDI_US_Moss (*n* = 6 (ref. ^36^)), MetS_NL_Koopen (*n* = 24 (ref. ^40^)), UC_US_Damman (*n* = 6 (ref.^43^)), UC_US_Nusbaum (*n* = 4 (ref. ^42^)), UC_US_Lee (*n* = 2 (ref. ^29^)), CD_US_Vaughn (*n* = 18 (ref. ^45^)), ABXR_div_Leo (*n* = 26 (ref. ^39^)), ABXR_IS_BarYoseph (*n* = 14 (ref. ^38^)), IBS_NO_Goll (*n* = 30 (ref. ^44^)), MEL_US_Davar (*n* = 27 (ref. ^10^)), MEL_US_Baruch (*n* = 10^9^), REN_IT_Ianiro (*n* = 10 (ref. ^46^)), TOU_CN_Zhao (*n* = 5 (ref. ^47^)) and CTR_RU_Goloshchapov (*n* = 3 (ref. ^48^)). Contextual data, including donor–recipient matchings and information about clinical response, were curated from the study publications and, in some cases, kindly amended by the studies’ original authors on request (Supplementary Tables 1–3).

### Metagenomic data processing and taxonomic and functional profiling

Metagenomic reads were quality trimmed to remove base calls with a Phred score of <25. Reads were then discarded if they were <45 nucleotides or if they mapped to the human genome (GRCh38.p10) with at least 90% identity over 45 nucleotides. This processing was performed using NGLess^62^. Taxonomic profiles per sample were obtained using mOTUs v.2 (ref. ^63^). For functional profiling, reads were mapped against the Global Microbial Gene Catalog v.1 gut subcatalogue (gmgc.embl.de^64^) with a minimum match length of 45 nucleotides with at least 97% identity, and summarized based on antimicrobial resistance gene (ARG) annotations and Kyoto Encyclopedia of Genes and Genomes orthologs (KOs) via eggNOG annotations^65^. Based on the resulting KO profiles, GMMs^66^ were quantified in each sample using omixer-rpmR (v.0.3.2)^67^. Taxonomic and GMM profiles per sample, normalized by read depth, are available in Supplementary Tables 7 and 8.

### MAGs

We demarcated MAGs from samples of studies MetS_NL_1, UC_NL, ABXR_NL, div_AU, RCDI_US_Smillie, RCDI_US_Moss, UC_US_Damman, UC_US_Nusbaum, UC_US_Lee and CD_US_Vaughn using several complementary strategies to obtain both high resolution from sample-specific assemblies and deep coverage of lowly abundant species from coassemblies of multiple samples. Unless otherwise indicated, all tools in the following were run with default parameters.

To generate single-sample MAGs, fecal metagenomes were assembled individually using metaSPAdes v.3.12.0 (ref. ^68^), reads were mapped back to contigs using bwa-mem v.0.7.17 (ref. ^69^) and contigs were binned using metaBAT v.2.12.1 (ref. ^70^). Multisample MAGs were built for each cohort separately. Reads were first coassembled using megahit v.1.1.3 (ref. ^71^) and mapped back to contigs using bwa-mem v.0.7.17. Coassembled contigs were then binned using both CONCOCT v.0.5.0 (ref. ^72^) and metaBAT v.2.12.1. The resulting coassembled MAG sets were further refined using DAS TOOL^73^ and metaWRAP^74^. In total, 47,548 MAGs were demarcated using these five approaches (single-sample MAGs, multisample coassembled CONCOCT, metaBAT2, DAS TOOL and metaWRAP MAGs). In addition, we included 25,037 high-quality reference genomes from the proGenomes database^75,76^ in downstream analyses.

Genome quality was estimated using CheckM^77^ and GUNC v.0.1 (ref. ^78^), and all genomes were taxonomically classified using GTDB-tk^79^. Open reading frames (ORFs) were predicted using prodigal^80^ and annotated via prokka workflow v.1.14.6 (ref. ^81^). Orthologs to known gene families were detected using eggNOG-mapper v.1 (ref. ^82^). ARGs were annotated using a workflow combining information from databases CARD v.3.0.0 (via rgi v.4.2.4 (ref. ^83^) and ResFams v.1.2.2 (ref. ^84^), as described previously^76^. The ‘specI’ set of 40 near-universal single-copy marker genes were detected in each genome using fetchMG^85^.

The full set of generated MAGs and contextual data are available via Zenodo (DOI 10.5281/zenodo.5534163 (ref. ^86^)).

### Genome clustering, species metapangenomes and phylogeny

Genomes were clustered into species-level groups using an ‘open-reference’ approach in multiple steps. Initial prefiltering using lenient quality criteria (CheckM-estimated completeness ≥70%, contamination ≤25%; additional criteria were applied downstream) removed 57.7% of MAGs. The remaining 20,093 MAGs were mapped to the clustered proGenomes v.1 (ref. ^75^) and mOTUs v.2 (ref. ^63^) taxonomic marker gene databases using MAPseq v.1.2.3 (ref. ^87^). A total of 17,720 MAGs were confidently assigned to a ref-mOTU (specI cluster) or meta-mOTU based on the following criteria: (1) detection of at least 20% of the screened taxonomic marker genes and (2) a majority of markers assigning to the same mOTU at a conservative MAPseq confidence threshold of ≥0.9.

In an independent approach, quality-filtered MAGs and reference genomes were also clustered by average nucleotide identity (ANI) using a modified and scalable reimplementation of the dRep workflow^88^. Using pairwise distances computed with mash v.2.1 (ref. ^89^), sequences were first preclustered to 90% mash-ANI using the single-linkage algorithm, asserting that all genome pairs sharing ≥90% mash-ANI were grouped together. Each mash precluster was then resolved to 95 and 99% average linkage ANI clusters using fastANI v.1.1 (ref. ^90^). For each cluster, a representative genome was picked as either the corresponding reference specI cluster representative in the proGenomes database or the MAG with the highest dRep score (calculated based on estimated completeness and contamination). Genome partitions based on 95% average linkage ANI clustering and specI marker gene mappings matched almost perfectly, at an adjusted Rand index of >0.99. We therefore defined a total of 1,089 species-level clusters (‘species’) from our dataset (Supplementary Table 4), primarily based on marker gene mappings to precomputed ref-mOTUs (or specI clusters, *n* = 295) and meta-mOTUs (*n* = 528), and as 95% average linkage ANI clusters for genomes that did not map to either of these databases (*n* = 233).

Species pangenomes were generated by clustering all genes within each species-level cluster at 95% amino acid identity, using Roary 3.12.0 (ref. ^91^). Spurious and putatively contaminant gene clusters (as introduced by misbinned contigs in MAGs) were removed by asserting that the underlying gene sequences originated (1) from a reference genome in the proGenomes database or (2) from at least two independent MAGs, assembled from distinct samples or studies. To account for incomplete genomes, ‘extended core genes’ were defined as gene clusters present in >80% of genomes in a species-level cluster. If too few gene clusters satisfied this criterion, as was the case for some pangenomes containing many incomplete MAGs, the 50 most prevalent gene clusters were used instead. Representative sequences for each gene cluster were picked as ORFs originating from specI representative genomes (that is, high-quality reference genomes), or otherwise as the longest ORF in the cluster.

A phylogenetic tree of species-level cluster representatives was inferred based on the ‘mOTU’ set of ten near-universal marker genes^63^. Marker genes were aligned in amino acid sequence space across all species using Muscle v.3.8.31 (ref. ^92^), concatenated and then used to construct a species tree with FastTree2 (v.2.1.11)^93^ with default parameters.

### Inference of microbial strain populations

Metagenomic reads for each sample were mapped against gene cluster representative sequences for all species pangenomes using bwa-mem v.0.7.17 (ref. ^69^). Mapped reads were filtered for matches of ≥45 bp and ≥97% sequence identity, sorted and filtered against multiple mappings using samtools v.1.7 (ref. ^94^). Horizontal (‘breadth’) and vertical (‘depth’) coverage of each gene cluster in each sample were calculated using bedtools v.2.27.1 (ref. ^95^).

A species was considered present in a sample if at least three mOTU taxonomic marker genes were confidently detected either via the mOTU v.2 profiler (for specI clusters and meta-mOTUs) or based on pangenome-wide read mappings (for non-mOTU species-level clusters). Gene clusters within each pangenome were considered present in a sample if (1) the species was detectable (see above), (2) horizontal coverage exceeded 100 bp and 20% of the representative gene’s length and (3) average vertical coverage exceeded 0.5. Gene clusters were considered confidently absent if they did not attract any mappings in samples where the species’ set of extended core genes (see above) was covered at >1 median vertical coverage (that is, present with high confidence). Using these criteria, strain population-specific gene content profiles were computed for each species in each sample.

Raw microbial SNVs were called from uniquely mapping reads using metaSNV v.1.0.3 (ref. ^96^) with permissive parameters (-c 10 -t 2 -p 0.001 -d 1000000). Candidate SNVs were retained if they were supported by two or more reads each in two or more samples in which the focal gene cluster was confidently detected (see above), before differential downstream filtering. At multiallelic positions the frequency of each observed allele (A, C, G, T) was normalized by the total read depth for all alleles.

Based on these data, strain populations were represented based on both their specific gene content profile and SNV profile in each sample.

Each species’ local strain population diversity (SPD) and allele distances (AD) between strain populations across samples were estimated as follows. SPD was calculated based on the inverse Simpson index of allele frequencies *p*_(ACGT)_ at each variant position *i* in the extended core genome (n_var_), normalized by total horizontal coverage (number of covered positions) cov_hor_:

$${\mathrm{SPD}} = \frac{{\mathop {\sum}\nolimits_{i = 1}^{n_{{\mathrm{var}}}} {\left( {p_{\mathrm{A}}^2 + p_{\mathrm{C}}^2 + p_{\mathrm{G}}^2 + p_{\mathrm{T}}^2} \right)^{ - 1} - 1} }}{{{\mathrm{cov}}_{{\mathrm{hor}}}}}$$

Thus defined, SPD can be interpreted as the average effective number of nondominant alleles in a strain population. SPD ranges between 0 (only one dominant strain detected—that is, no multiallelic positions) and 3 (all four possible alleles present at equal proportions at each variant position). Normalization by total horizontal coverage, cov_hor_ of the extended core genome ensures that values are comparable between samples even if a species’ coverage in a sample is incomplete.

Intraspecific ADs between strain populations across samples were calculated as the average Euclidean distance between observed allele frequencies at variant positions in the species’ extended core genome, requiring at least 20 variant positions with shared coverage between samples. If a species was not observed in a sample, ADs to that sample were set to 1.

### Quantification of strain-level outcomes

Colonization by donor strains, persistence of recipient strains and influx of novel strains (environmental or previously below detection limit) in the recipient microbiome following FMT were quantified for every species based on determinant microbial SNVs and gene content profiles using an approach extending previous work^25,97^. In total, 261 FMT time series (228 allogenic and 33 autologous transfers) for which a donor baseline (in allogenic FMTs; ‘D’), a recipient pre-FMT baseline (‘R’) and at least one recipient post-FMT (‘P’) sample were available were taken into account, and each FMT was represented as a D-R-P sample triad. If available, multiple time points post FMT were scored independently. By definition, because no donor samples were available for autologous FMTs, recipient pre-FMT samples were used instead. An overview of potential strain-level FMT outcomes is provided in Fig. 1c,d.

For each D-R-P sample triad, conspecific strain dynamics were calculated if a species was observed in all three samples (see above) with at least 100 informative (determinant) variant positions either covered with two or more reads or confidently absent (see below). Donor determinant alleles were defined as variants unique to the donor (D) relative to the recipient pre-FMT (R) sample, and vice versa. Post-FMT determinant alleles were defined as variants unique in P relative to both D and R. Given that intraspecific fecal strain populations are often heterogeneous—that is, consist of more than one strain per species—multiple observed alleles at the same variant position were taken into account. In addition, if a gene containing a putative variant position was absent from a sample although the species’ extended core genome was detected, the variant was considered ‘confidently absent’ and treated as informative (and potentially determinant) as well, thereby taking into account differential gene content between strains.

The fractions of donor and recipient strains post FMT were quantified based on the detection of donor- and recipient-determinant variants across all informative positions in the P sample. The fraction of novel strains (environmental or previously below detection limit in donor and recipient) was quantified as the fraction of post-FMT determinant variants. Based on these three readouts (fraction of donor, recipient and novel strains) and cutoffs previously established by Li et al.^25^, FMT outcomes were scored categorically as ‘donor colonization’, ‘recipient persistence’, ‘donor–recipient coexistence’ or ‘influx of novel (previously undetected) strains’ for every species (Supplementary Table 5).

In addition to conspecific strain dynamics (that is, where a species was present in D, R and P), we also quantified FMT outcomes that involved the acquisition or loss of entire strain populations. For example, if a species was present in the recipient at baseline but not post FMT, this was considered a ‘species loss’ event. See Fig. 1c and Supplementary Table 5 for a full overview of how different FMT outcome scenarios were scored.

To assert the accuracy of our approach, we simulated FMT time series by shuffling (1) the donor sample, (2) the recipient pre-FMT sample or (3) both. Randomizations were stratified by subject (accounting for the fact that some donors were used in multiple FMTs and that some recipients received repeated treatments) and geography. For each observed D-R-P sample triad, we simulated ten triads per each of the above setups.

Outcomes were further summarized across species by calculating a series of strain population-level metrics for each FMT, defined as follows.

Persistence index: average fraction of persistent recipient strains among all species observed post FMT (that is, fraction of post-FMT strain populations attributable to recipient baseline strains).

Colonization index: average fraction of donor strains among all species post FMT.

### Modeling and prediction of FMT outcomes

We explored a large set of covariates as putative predictor variables for FMT outcomes, grouped into the following categories: (1) host clinical and procedural variables (for example, FMT indication, pre-FMT bowel preparation, FMT route and so on); (2) community-level taxonomic diversity (species richness, community composition and so on); (3) community-level metabolic profiles (abundance of specific pathways); (4) abundance profiles of individual species; (5) strain-level outcomes for other species in the system; and (6) focal species characteristics, including strain-level diversity; see Supplementary Table 6 for a full list of covariates and their definitions. We further classified covariates as either predictive ex ante variables (that is, knowable before the FMT is conducted) or post hoc variables (that is, pertaining to the post-FMT state, or the relation between pre- and post-FMT states).

We built two types of model to predict FMT strain-level outcomes based on these covariates: (1) FMT-wide models, using summary outcome metrics across all species in a time series (persistence index, colonization index; see above) as response variables; and (2) per-species models for 307 species observed in ≥50 FMTs, using each species’ strain-level outcome in every scored time series as response variable. Unless otherwise indicated, the last available time point for each FMT time series was used. Models were built for each covariate category separately, as well as for combinations of all ex ante and all post hoc variables, respectively.

Given that the number of covariates greatly exceeded the number of available FMT time series, and that several covariates were correlated with each other (Supplementary Fig. 3), FMT outcomes were modeled using ten times fivefold cross-validated LASSO-regularized regression, as implemented in the R package glmnet (v.4.1.3)^98^. Regression coefficients were chosen at one standard error from the cross-validated minimum lambda value and averaged across validation folds.

Linear LASSO regression was used to model outcomes with continuous response variables, both for FMT-wide outcomes (persistence index and soon) and for the fraction of colonizing, persisting and coexisting strains per species across FMTs. For linear models, *R*^2^ of predictions on test sets was averaged across validation folds. Moreover, logistic LASSO regression was used to additionally model binarized FMT outcomes per species, defined as recipient strain resilience, recipient strain turnover and donor strain takeover, based on further summarizing outcome categories in Supplementary Table 5. For logistic models, accuracy was assessed as area under the receiver operating characteristic curve (AUROC) averaged across validation folds.

### Statistical analyses

Association of clinical outcomes (excluding a subset of cohorts for which clinical success was not reported; Supplementary Table 3) with FMT strain-level outcomes was tested using Wilcoxon tests (responders versus nonresponders), and also by sequential ANOVA on linear regression models (accounting for additional variables), in each case followed by Benjamini–Hochberg correction for multiple hypothesis tests. Differences in strain-level outcomes between species across taxonomic clades and inferred species phenotypes were tested using ANOVA on linear regression models.

### Reporting summary

Further information on research design is available in the Nature Research Reporting Summary linked to this article.

## Online content

Any methods, additional references, Nature Research reporting summaries, source data, extended data, supplementary information, acknowledgements, peer review information; details of author contributions and competing interests; and statements of data and code availability are available at 10.1038/s41591-022-01913-0.

## Supplementary information

Supplementary Information Supplementary Figs. 1–3.

Reporting Summary

Supplementary Tables Supplementary Tables 1–8.

## Data Availability

Raw metagenomic sequencing data have been uploaded to the European Nucleotide Archive under accession nos. PRJEB46777, PRJEB46778, PRJEB46779 and PRJEB46780. The full list of included publicly available datasets, including accession numbers and associated PMIDs, is available in Supplementary Table 1. Contextual data are available in Supplementary Tables 2 and 3. MAGs (10.5281/zenodo.5534163)^86^ and source data (10.5281/zenodo.6611040)^99^ are available for download via Zenodo.

## Code availability

Analysis code is available via github (https://github.com/grp-bork/fmt_metastudy).

## References

[1] Borody TJ (2004). Bacteriotherapy using fecal flora: toying with human motions. J. Clin. Gastroenterol..

[2] Rossen NG (2015). Fecal microbiota transplantation as novel therapy in gastroenterology: a systematic review. World J. Gastroenterol..

[3] Hanssen NMJ, de Vos WM, Nieuwdorp M (2021). Fecal microbiota transplantation in human metabolic diseases: from a murky past to a bright future?. Cell Metab..

[4] Gough E, Shaikh H, Manges AR (2011). Systematic review of intestinal microbiota transplantation (fecal bacteriotherapy) for recurrent *Clostridium difficile* infection. Clin. Infect. Dis..

[5] van Nood E (2013). Duodenal infusion of donor feces for recurrent *Clostridium difficile*. N. Engl. J. Med..

[6] Narula N (2017). Systematic review and meta-analysis fecal microbiota transplantation for treatment of active ulcerative colitis. Inflamm. Bowel Dis..

[7] Haifer C, Leong RW, Paramsothy S (2020). The role of fecal microbiota transplantation in the treatment of inflammatory bowel disease. Curr. Opin. Pharmacol..

[8] Suez J (2018). Post-antibiotic gut mucosal microbiome reconstitution is impaired by probiotics and improved by autologous FMT. Cell.

[9] Baruch, E. N. et al. Fecal microbiota transplant promotes response in immunotherapy-refractory melanoma patients. *Science* **371**, 602–609 (2021). 10.1126/science.abb5920 33303685

[10] Davar D (2021). Fecal microbiota transplant overcomes resistance to anti-PD-1 therapy in melanoma patients. Science.

[11] Burrello C (2018). Therapeutic fecal microbiota transplantation controls intestinal inflammation through IL10 secretion by immune cells. Nat. Commun..

[12] Seekatz AM (2018). Restoration of short chain fatty acid and bile acid metabolism following fecal microbiota transplantation in patients with recurrent *Clostridium difficile* infection. Anaerobe.

[13] Zuo T (2017). Bacteriophage transfer during fecal microbiota transplantation is associated with treatment response in *Clostridium difficile* infection. Gastroenterology.

[14] Manrique P (2021). Gut bacteriophage dynamics during fecal microbial transplantation in subjects with metabolic syndrome. Gut Microbes.

[15] Wilson BC, Vatanen T, Cutfield WS, O’Sullivan JM (2019). The super-donor phenomenon in fecal microbiota transplantation. Front. Cell. Infect. Microbiol..

[16] Duvallet, C. et al. Framework for rational donor selection in fecal microbiota transplant clinical trials. *PloS ONE* **14**, e0222881 (2019). 10.1371/journal.pone.0222881 PMC6786724 31600222

[17] Olesen SW, Gerardin Y (2021). Re-evaluating the evidence for fecal microbiota transplantation “super-donors” in inflammatory bowel disease. J. Crohns Colitis.

[18] Kootte RS (2017). Improvement of insulin sensitivity after lean donor feces in metabolic syndrome is driven by baseline intestinal imcrobiota composition. Cell Metab..

[19] Danne, C., Rolhion, N. & Sokol, H. Recipient factors in fecal microbiota transplantation: one stool does not fit all. *Nat. Rev. Gastroenterol. Hepatol*. **18**, 503–513 (2021). 10.1038/s41575-021-00441-5 33907321

[20] Fujimoto K (2021). Functional restoration of bacteriomes and viromes by fecal microbiota transplantation. Gastroenterology.

[21] Peri R (2019). The impact of technical and clinical factors on fecal microbiota transfer outcomes for the treatment of recurrent *Clostridioides difficile* infections in Germany. United European Gastroenterol. J..

[22] Draper LA (2018). Long-term colonisation with donor bacteriophages following successful fecal microbial transplantation. Microbiome.

[23] Leonardi, I. et al. Fungal trans-kingdom dynamics linked to responsiveness to fecal microbiota transplantation (FMT) therapy in ulcerative colitis. *Cell Host Microbe* **27**, 823–829 (2020). 10.1016/j.chom.2020.03.006 PMC8647676 32298656

[24] Zuo T (2018). Gut fungal dysbiosis correlates with reduced efficacy of fecal microbiota transplantation in *Clostridium difficile* infection. Nat. Commun..

[25] Li SS (2016). Durable coexistence of donor and recipient strains after fecal microbiota transplantation. Science.

[26] Smillie CS (2018). Strain tracking reveals the determinants of bacterial engraftment in the human gut following fecal microbiota transplantation. Cell Host Microbe.

[27] Podlesny D (2022). Metagenomic strain detection with SameStr: identification of a persisting core gut microbiota transferable by fecal transplantation. Microbiome.

[28] Aggarwala V (2021). Precise quantification of bacterial strains after fecal microbiota transplantation delineates long-term engraftment and explains outcomes. Nat. Microbiol..

[29] Lee STM (2017). Tracking microbial colonization in fecal microbiota transplantation experiments via genome-resolved metagenomics. Microbiome.

[30] Wilson BC (2021). Strain engraftment competition and functional augmentation in a multi-donor fecal microbiota transplantation trial for obesity. Microbiome.

[31] Ng SC (2022). Microbiota engraftment after fecal microbiota transplantation in obese subjects with type 2 diabetes: a 24-week, double-blind, randomised controlled trial. Gut.

[32] Watson, A. R., Fuessel, J., Veseli, I. & DeLongchamp, J. Z. Adaptive ecological processes and metabolic independence drive microbial colonization and resilience in the human gut. Preprint at 10.1101/2021.03.02.433653 (2021).

[33] Walter J, Maldonado-Gómez MX, Martínez I (2018). To engraft or not to engraft: an ecological framework for gut microbiome modulation with live microbes. Curr. Opin. Biotechnol..

[34] Schmidt TSB, Raes J, Bork P (2018). The human gut microbiome: from association to modulation. Cell.

[35] Xiao Y, Angulo MT, Lao S, Weiss ST, Liu Y-Y (2020). An ecological framework to understand the efficacy of fecal microbiota transplantation. Nat. Commun..

[36] Moss EL (2017). Long-term taxonomic and functional divergence from donor bacterial strains following fecal microbiota transplantation in immunocompromised patients. PLoS ONE.

[37] Singh R (2018). Fecal microbiota transplantation against intestinal colonization by extended spectrum beta-lactamase producing Enterobacteriaceae: a proof of principle study. BMC Res. Notes.

[38] Bar-Yoseph, H. et al. Oral capsulized fecal microbiota transplantation for eradication of carbapenemase-producing Enterobacteriaceae colonization with a metagenomic perspective. *Clin. Infect. Dis*. **73**, e166–e175 (2020). 10.1093/cid/ciaa737 32511695

[39] Leo, S. et al. Metagenomic characterization of gut microbiota of carriers of extended-spectrum beta-lactamase or carbapenemase-producing Enterobacteriaceae following treatment with oral antibiotics and fecal microbiota transplantation: results from a multicenter randomized trial. *Microorganisms* **8**, 941 (2020). 10.3390/microorganisms8060941 PMC7357103 32585945

[40] Koopen AM (2021). Effect of fecal microbiota transplantation combined with Mediterranean diet on insulin sensitivity in subjects with metabolic syndrome. Front. Microbiol..

[41] Rossen NG (2015). Findings from a randomized controlled trial of fecal transplantation for patients with ulcerative colitis. Gastroenterology.

[42] Nusbaum, D. J. et al. Gut microbial and metabolomic profiles after fecal microbiota transplantation in pediatric ulcerative colitis patients. *FEMS Microbiol. Ecol*. **94**, fiy133 (2018). 10.1093/femsec/fiy133 PMC6454419 30010747

[43] Damman CJ (2015). Low level engraftment and improvement following a single colonoscopic administration of fecal microbiota to patients with ulcerative colitis. PLoS ONE.

[44] Goll R (2020). Effects of fecal microbiota transplantation in subjects with irritable bowel syndrome are mirrored by changes in gut microbiome. Gut Microbes.

[45] Vaughn BP (2016). Increased intestinal microbial diversity following fecal microbiota transplant for active Crohn’s disease. Inflamm. Bowel Dis..

[46] Ianiro G (2020). Fecal microbiota transplantation for the treatment of diarrhoea induced by tyrosine-kinase inhibitors in patients with metastatic renal cell carcinoma. Nat. Commun..

[47] Zhao H-J (2020). The efficacy of fecal microbiota transplantation for children with Tourette syndrome: a preliminary study. Front. Psychiatry.

[48] Goloshchapov OV (2019). Long-term impact of fecal transplantation in healthy volunteers. BMC Microbiol..

[49] Tian L (2020). Deciphering functional redundancy in the human microbiome. Nat. Commun..

[50] Hildebrand F (2021). Dispersal strategies shape persistence and evolution of human gut bacteria. Cell Host Microbe.

[51] Lahti L, Salojärvi J, Salonen A, Scheffer M, de Vos WM (2014). Tipping elements in the human intestinal ecosystem. Nat. Commun..

[52] Scheffer, M., Carpenter, S. R., Dakos, V. & van Nes, E. H. Generic indicators of ecological resilience: inferring the chance of a critical transition. *Annu. Rev. Ecol. Evol. Syst*. **46**, 145–167 (2015).

[53] Gonze D, Lahti L, Raes J, Faust K (2017). Multi-stability and the origin of microbial community types. ISME J..

[54] Costea PI (2018). Enterotypes in the landscape of gut microbial community composition. Nat. Microbiol..

[55] Debray, R. et al. Priority effects in microbiome assembly. *Nat. Rev. Microbiol*. **20**, 109–121 (2021). 10.1038/s41579-021-00604-w 34453137

[56] Zaneveld JR, McMinds R, Thurber RV (2017). Stress and stability: applying the Anna Karenina principle to animal microbiomes. Nat. Microbiol..

[57] Basson AR, Zhou Y, Seo B, Rodriguez-Palacios A, Cominelli F (2020). Autologous fecal microbiota transplantation for the treatment of inflammatory bowel disease. Transl. Res..

[58] de Groot P (2021). Fecal microbiota transplantation halts progression of human new-onset type 1 diabetes in a randomised controlled trial. Gut.

[59] Ott SJ (2017). Efficacy of sterile fecal filtrate transfer for treating patients with *Clostridium difficile* infection. Gastroenterology.

[60] Bojanova DP, Bordenstein SR (2016). Fecal transplants: what is being transferred?. PLoS Biol..

[61] Fuentes S (2017). Microbial shifts and signatures of long-term remission in ulcerative colitis after fecal microbiota transplantation. ISME J..

[62] Coelho LP (2019). NG-meta-profiler: fast processing of metagenomes using NGLess, a domain-specific language. Microbiome.

[63] Milanese A (2019). Microbial abundance, activity and population genomic profiling with mOTUs2. Nat. Commun..

[64] Coelho LP (2022). Towards the biogeography of prokaryotic genes. Nature.

[65] Huerta-Cepas J (2019). EggNOG 5.0: a hierarchical, functionally and phylogenetically annotated orthology resource based on 5090 organisms and 2502 viruses. Nucleic Acids Res..

[66] Vieira-Silva S (2016). Species–function relationships shape ecological properties of the human gut microbiome. Nat. Microbiol..

[67] Darzi Y, Falony G, Vieira-Silva S, Raes J (2016). Towards biome-specific analysis of meta-omics data. ISME J..

[68] Nurk S, Meleshko D, Korobeynikov A, Pevzner PA (2017). MetaSPAdes: a new versatile metagenomic assembler. Genome Res..

[69] Li, H. Aligning sequence reads, clone sequences and assembly contigs with BWA-MEM. Preprint at 10.48550/arXiv.1303.3997 (2013).

[70] Kang DD (2019). MetaBAT 2: an adaptive binning algorithm for robust and efficient genome reconstruction from metagenome assemblies. PeerJ..

[71] Li D, Liu C-M, Luo R, Sadakane K, Lam T-W (2015). MEGAHIT: an ultra-fast single-node solution for large and complex metagenomics assembly via succinct de Bruijn graph. Bioinformatics.

[72] Alneberg J (2014). Binning metagenomic contigs by coverage and composition. Nat. Methods.

[73] Sieber CMK (2018). Recovery of genomes from metagenomes via a dereplication, aggregation and scoring strategy. Nat. Microbiol..

[74] Uritskiy GV, DiRuggiero J, Taylor J (2018). MetaWRAP—a flexible pipeline for genome-resolved metagenomic data analysis. Microbiome.

[75] Mende DR (2017). ProGenomes: a resource for consistent functional and taxonomic annotations of prokaryotic genomes. Nucleic Acids Res..

[76] Mende DR (2020). ProGenomes2: an improved database for accurate and consistent habitat, taxonomic and functional annotations of prokaryotic genomes. Nucleic Acids Res..

[77] Parks DH, Imelfort M, Skennerton CT, Hugenholtz P, Tyson GW (2015). CheckM: assessing the quality of microbial genomes recovered from isolates, single cells, and metagenomes. Genome Res..

[78] Orakov A (2021). GUNC: detection of chimerism and contamination in prokaryotic genomes. Genome Biol..

[79] Chaumeil, P.-A., Mussig, A. J., Hugenholtz, P. & Parks, D. H. GTDB-Tk: a toolkit to classify genomes with the Genome Taxonomy Database. *Bioinformatics* **36**, 1925–1927 (2019). 10.1093/bioinformatics/btz848 PMC7703759 31730192

[80] Hyatt D (2010). Prodigal: prokaryotic gene recognition and translation initiation site identification. BMC Bioinformatics.

[81] Seemann T (2014). Prokka: rapid prokaryotic genome annotation. Bioinformatics.

[82] Huerta-Cepas J (2017). Fast genome-wide functional annotation through orthology assignment by eggNOG-Mapper. Mol. Biol. Evol..

[83] Alcock BP (2020). CARD 2020: antibiotic resistome surveillance with the comprehensive antibiotic resistance database. Nucleic Acids Res..

[84] Gibson MK, Forsberg KJ, Dantas G (2015). Improved annotation of antibiotic resistance determinants reveals microbial resistomes cluster by ecology. ISME J..

[85] Mende DR, Sunagawa S, Zeller G, Bork P (2013). Accurate and universal delineation of prokaryotic species. Nat. Publ. Group.

[86] Schmidt, T. S. B. et al. Drivers and determinants of strain dynamics following fecal microbiota transplantation. *Zenodo*10.5281/ZENODO.5534163 (2021). 10.1038/s41591-022-01913-0 PMC9499871 36109636

[87] Rodrigues, J. F. M., Schmidt, S. B. T., Tackmann, J. & von Mering, C. MAPseq: highly efficient k-mer search with confidence estimates, for rRNA sequence analysis. *Bioinformatics* **33**, 3808–3810 (2017). 10.1093/bioinformatics/btx517 PMC5860325 28961926

[88] Olm MR, Brown CT, Brooks B, Banfield JF (2017). DRep: a tool for fast and accurate genomic comparisons that enables improved genome recovery from metagenomes through de-replication. ISME J..

[89] Ondov BD (2016). Mash: fast genome and metagenome distance estimation using MinHash. Genome Biol..

[90] Jain C, Rodriguez-R LM, Phillippy AM, Konstantinidis KT, Aluru S (2018). High throughput ANI analysis of 90 K prokaryotic genomes reveals clear species boundaries. Nat. Commun..

[91] Page AJ (2015). Roary: rapid large-scale prokaryote pan genome analysis. Bioinformatics.

[92] Edgar RC (2004). MUSCLE: a multiple sequence alignment method with reduced time and space complexity. BMC Bioinformatics.

[93] Price MN, Dehal PS, Arkin AP (2010). FastTree 2–approximately maximum-likelihood trees for large alignments. PLoS ONE.

[94] Li H (2009). The Sequence Alignment/Map format and SAMtools. Bioinformatics.

[95] Quinlan AR, Hall IM (2010). BEDTools: a flexible suite of utilities for comparing genomic features. Bioinformatics.

[96] Costea PI (2017). MetaSNV: a tool for metagenomic strain level analysis. PLoS ONE.

[97] Schmidt, T. S. B. et al. Extensive transmission of microbes along the gastrointestinal tract. *eLife* **8**, e42693 (2019). 10.7554/eLife.42693 PMC6424576 30747106

[98] Friedman J, Hastie T, Tibshirani R (2010). Regularization paths for generalized linear models via coordinate descent. J. Stat. Softw..

[99] Schmidt, T. S. B. et al. Analysis data, “Drivers and Determinants of Strain Dynamics Following Fecal Microbiota Transplantation”. *Zenodo*10.5281/ZENODO.5534163 (2021). 10.1038/s41591-022-01913-0 PMC9499871 36109636

