# Supplementary material for: Drivers and determinants of strain dynamics following fecal microbiota transplantation
Source: Nat Med. 2022 Sep 15;28(9):1902–12. doi: 10.1038/s41591-022-01913-0 (PMC9499871; doi:10.1038/s41591-022-01913-0)
Supplement: Supplementary file 1 — Supplementary Figs. 1–3. [file 41591_2022_1913_MOESM1_ESM.pdf]

nature portfolio

https://doi.org/10.1038/s41591-022-01913-0

Supplementary information

Drivers and determinants of strain dynamics following fecal microbiota transplantation

In the format provided by the
authors and unedited

Figure S1. Strain-level outcomes for 307 prevalent species tracked across FMT time series. FMTs (columns) are organized by indication, scope (allogenic vs autologous) and clinical success. Species (rows) are organized by underlying phylogeny, corresponding to what is shown in Fig 4.

Figure S2. Ternary plots of the post-FMT strain population space for each of 307 prevalent species. Each dot corresponds to a *conspecific* FMT outcome for the focal species; non-conspecific outcomes (acquisition or loss of entire strain population, complete failure to colonize) are not shown. This is an extension to Fig 4A which shows a subset of these plots.

Figure S3. Collinearity of relevant predictor variables. The heatmap shows pairwise Spearman correlation values between candidate predictor variables, organized by type, scope & resolution as outlined in table S6.

Note: *supplementary tables are included in the submission as an xlsx file as 'source data' in order to be editable and accessible. Tables S1, S5 & S6 are also included in this PDF for reference. The data tables (S2, S3, S4, S7, S8) are moreover part of the source datasets deposited on Zenodo (see main text).*

Table S1. Overview of datasets used in this study.

Table S2. Contextual information for 295 FMT time series.

Table S3. Contextual information for all 1,492 analyzed fecal metagenome samples (with information on technical replicates).

Table S4. Contextual data for 1,089 species included in this study.

Table S5. Strain-level FMT outcome scoring rules, based on determinant microbial SNVs and gene content per species pan-genome in sample triads of donor baseline, recipient baseline and recipient post-FMT. See Methods and Fig 1C for details and visualization.

Table S6. Overview and definitions of variables tested for association with FMT outcomes. Variables are categorized by type, scope (pertaining to the host or microbiome, to the donor or recipient) and resolution (host-level, community-level, species-level, strain-level). See Methods for additional details.

Table S7. mOTU taxonomic profiles.

Table S8. GMM profiles.

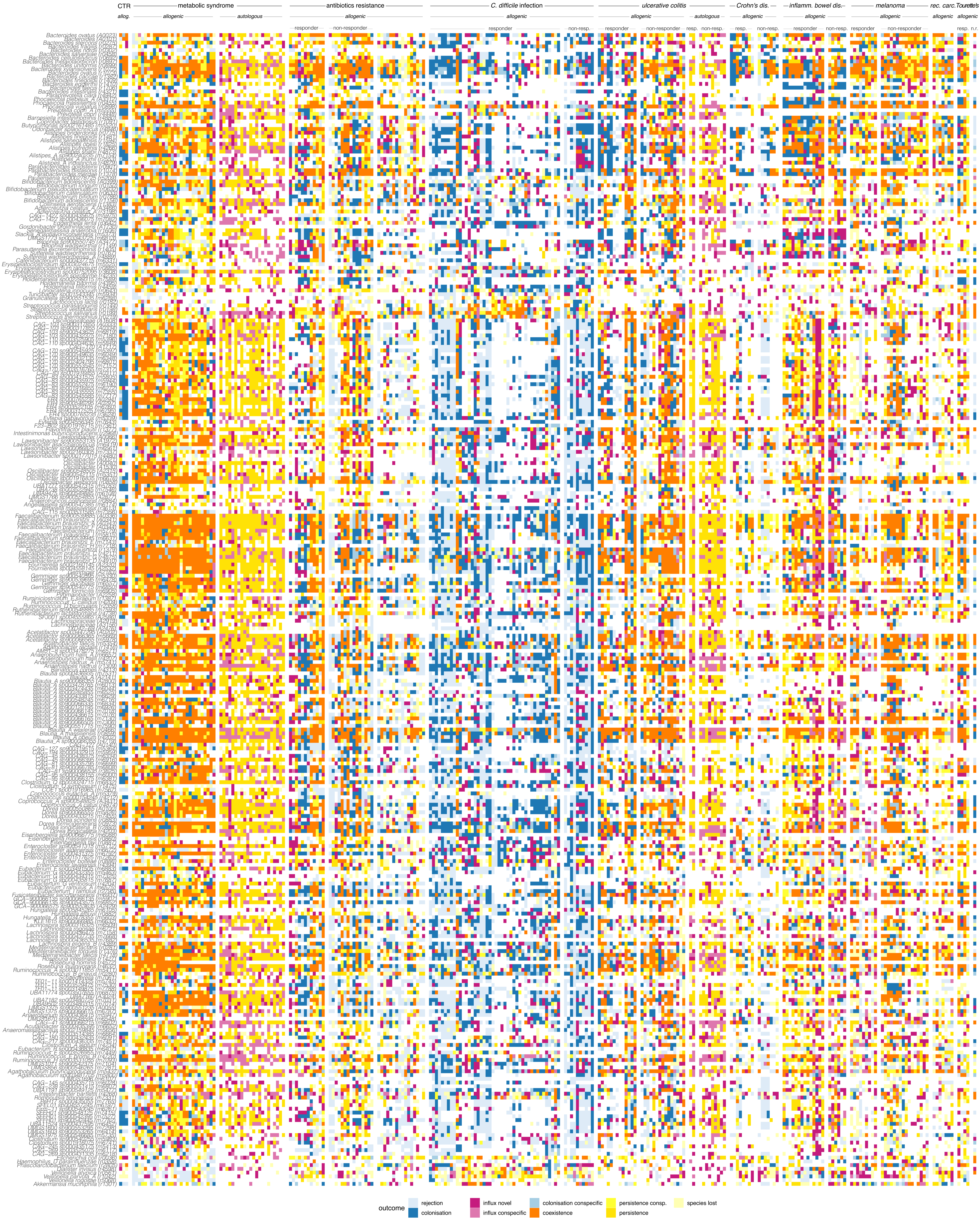

CTR
metabolic syndrome
antibiotics resistance
C. difficile infection
ulcerative colitis
Crohn's dis.
inflamm. bowel dis.
melanoma
rec. carc.
Tourette's
allog.
allogenic
autologous
responder
non-responder
non-resp.
resp.
n.r.
outcome
rejection
colonisation
influx novel
influx conspecific
colonisation conspecific
coexistence
persistence consp.
persistence
species lost

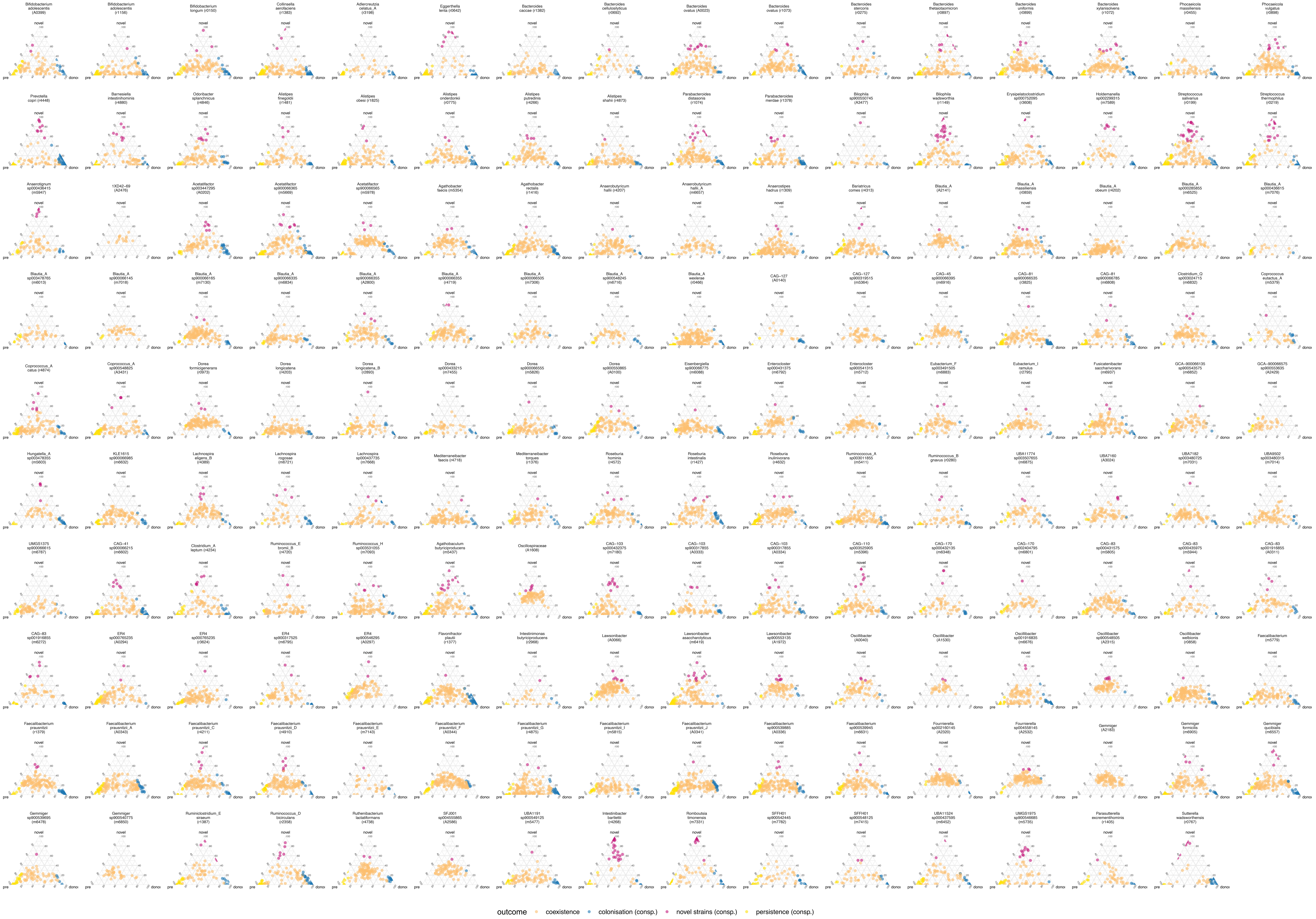
Bifidobacterium adolescentis (A0399)
Bifidobacterium adolescentis (r1156)
Bifidobacterium longum (r0150)
Collinsella aerofaciens (r1383)
Adlercreutzia celatus_A (r3198)
Eggerthella lenta (r0642)
Bacteroides caccae (r1382)
Bacteroides cellulosilyticus (r0692)
Bacteroides ovatus (A0023)
Bacteroides ovatus (r1073)
Bacteroides stercoris (r0275)
Bacteroides thetaiotaomicron (r0897)
Bacteroides uniformis (r0899)
Bacteroides xylanisolvens (r1072)
Phocaeicola massiliensis (r0455)
Phocaeicola vulgatus (r0898)
Prevotella copri (r4448)
Barnesiella intestinihominis (r4880)
Odoribacter splanchnicus (r4846)
Alistipes finegoldii (r1481)
Alistipes obesi (r1825)
Alistipes onderdonkii (r0775)
Alistipes putredinis (r4266)
Alistipes shahii (r4873)
Parabacteroides distasonis (r1074)
Parabacteroides merdae (r1378)
Bilophila sp900550745 (A3477)
Bilophila wadsworthia (r1149)
Erysipelatoclostridium sp000752095 (r3608)
Holdemanella sp002299315 (r7589)
Streptococcus salivarius (r0199)
Streptococcus thermophilus (r0219)
Anaerotignum sp000436415 (m5947)
1XD42−69 (A2476)
Acetatifactor sp003447295 (A0202)
Acetatifactor sp900066365 (m5669)
Acetatifactor sp900066565 (m5978)
Agathobacter faecis (m5354)
Agathobacter rectalis (r1416)
Anaerobutyricum hallii (r4207)
Anaerobutyricum hallii_A (m6657)
Anaerostipes hadrus (r1309)
Bariatricus comes (r4313)
Blautia_A (A2141)
Blautia_A massiliensis (r0859)
Blautia_A obeum (r4202)
Blautia_A sp000285855 (m6525)
Blautia_A sp000436615 (m7076)
Blautia_A sp003478765 (m6013)
Blautia_A sp900066145 (m7018)
Blautia_A sp900066165 (m7130)
Blautia_A sp900066335 (m6834)
Blautia_A sp900066355 (A2800)
Blautia_A sp900066355 (r4719)
Blautia_A sp900066505 (m7306)
Blautia_A sp900548245 (m6716)
Blautia_A wexlerae (r0466)
CAG−127 (A0140)
CAG−127 sp900319515 (m6364)
CAG−45 sp900066395 (m6916)
CAG−81 sp900066535 (r3825)
CAG−81 sp900066785 (m6832)
Clostridium_Q sp003024715 (m5379)
Coprococcus eutactus_A (r4516)
Coprococcus_A catus (r4874)
Coprococcus_A sp900548825 (A3431)
Dorea formicigenerans (r0973)
Dorea longicatena (r4203)
Dorea longicatena_B (r2893)
Dorea sp000433215 (m7455)
Dorea sp900066555 (m5826)
Dorea sp900550865 (A0100)
Eisenbergiella sp900066775 (m6088)
Enterocloster sp000431375 (m6792)
Enterocloster sp900541315 (m5712)
Eubacterium_F sp000431505 (m6883)
Eubacterium_I ramulus (r2795)
Fusicatenibacter saccharivorans (m6937)
GCA−900066135 sp900066135 (m6852)
GCA−900066575 sp900553635 (A2429)
Hungatella_A sp003478355 (m5603)
KLE1615 sp900066985 (m6632)
Lachnospira eligens_B (r4389)
Lachnospira rogosae (m6721)
Lachnospira sp000437735 (m7668)
Mediterraneibacter faecis (r4718)
Mediterraneibacter torques (r1376)
Roseburia hominis (r4572)
Roseburia intestinalis (r1427)
Roseburia inulinivorans (r4632)
Ruminococcus_A sp003011855 (m5411)
Ruminococcus_B gnavus (r0280)
UBA11774 sp003507655 (m6875)
UBA7160 (A3024)
UBA7182 sp003480725 (m7031)
UBA9502 sp900540315 (m7014)
UMGS1375 sp900066615 (m6787)
CAG−41 sp900066215 (m6602)
Clostridium_A leptum (r4234)
Ruminococcus_E bromii_B (r4720)
Ruminococcus_H sp003531055 (m7093)
Agathobaculum butyriciproducens (m5437)
Oscillospiraceae (A1608)
CAG−103 sp000432375 (m7180)
CAG−103 sp900317855 (A0333)
CAG−103 sp900317855 (A0334)
CAG−110 sp003525905 (m5396)
CAG−170 sp000432135 (m6348)
CAG−170 sp002404795 (m6801)
CAG−83 sp000431575 (m5805)
CAG−83 sp000435975 (m5944)
CAG−83 sp001916855 (A0311)
CAG−83 sp001916855 (m6272)
ER4 sp000765235 (A0294)
ER4 sp000765235 (r3624)
ER4 sp900317525 (m6795)
ER4 sp900546295 (A0297)
Flavonifractor plautii (r1377)
Intestinimonas butyriciproducens (r2968)
Lawsonibacter (A0066)
Lawsonibacter asaccharolyticus (m6419)
Lawsonibacter sp900553135 (A1972)
Oscillibacter (A0040)
Oscillibacter (A1530)
Oscillibacter sp001916835 (m6676)
Oscillibacter sp900548505 (A2315)
Oscillibacter welbionis (r0858)
Faecalibacterium (m5779)
Faecalibacterium prausnitzii (r1379)
Faecalibacterium prausnitzii_A (A0343)
Faecalibacterium prausnitzii_C (r4211)
Faecalibacterium prausnitzii_D (r4910)
Faecalibacterium prausnitzii_E (r7143)
Faecalibacterium prausnitzii_F (A0344)
Faecalibacterium prausnitzii_G (r4875)
Faecalibacterium prausnitzii_I (m5815)
Faecalibacterium prausnitzii_J (A0341)
Faecalibacterium sp900539885 (A0336)
Faecalibacterium sp900539945 (m6631)
Fournierella sp002160145 (A2320)
Fournierella sp004558145 (A2532)
Gemmiger (A2183)
Gemmiger formicilis (m6905)
Gemmiger qucibialis (m6557)
Gemmiger sp900539695 (m6478)
Gemmiger sp900540775 (m6850)
Ruminiclostridium_E siraeum (r1387)
Ruminococcus_D bicirculans (r2358)
Ruthenibacterium lactatiformans (r4738)
SFJ001 sp004555865 (A2586)
UBA1191 sp900549125 (m5477)
Intestinibacter bartlettii (r4268)
Romboutsia timonensis (m7331)
SFFH01 sp900542445 (m7782)
SFFH01 sp900548125 (m7415)
UBA11524 sp000437595 (m6452)
UMGS1975 sp900546685 (m5735)
Parasutterella excrementihominis (r1405)
Sutterella wadsworthensis (r0767)
novel
pre
donor
outcome
coexistence
colonisation (consp.)
novel strains (consp.)
persistence (consp.)

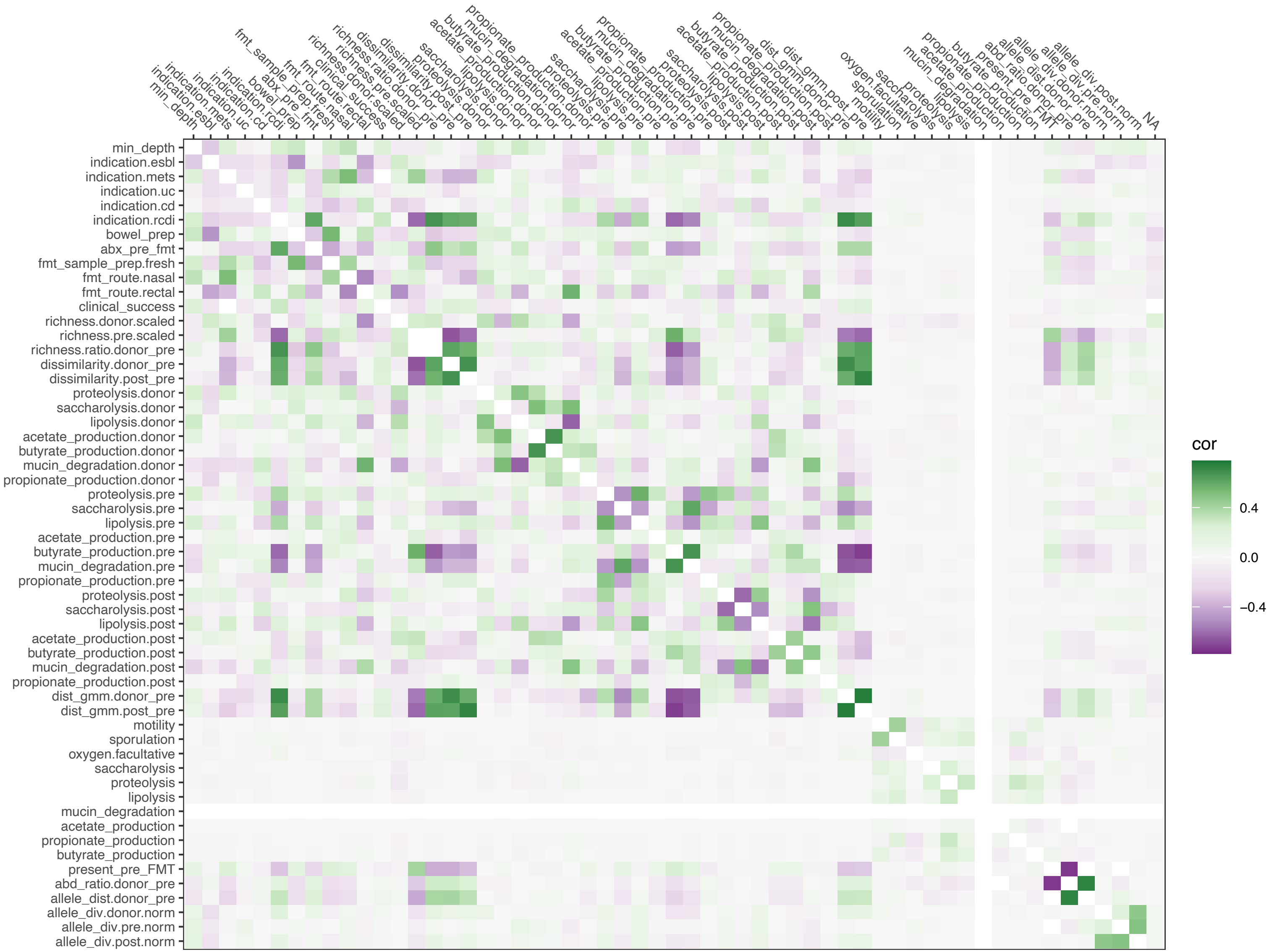
min_depth
indication.esbl
indication.mets
indication.uc
indication.cd
indication.rcdi
bowel_prep
abx_pre_fmt
fmt_sample_prep.fresh
fmt_route.nasal
fmt_route.rectal
clinical_success
richness.donor.scaled
richness.pre.scaled
richness.ratio.donor_pre
dissimilarity.donor_pre
dissimilarity.post_pre
proteolysis.donor
saccharolysis.donor
lipolysis.donor
acetate_production.donor
butyrate_production.donor
mucin_degradation.donor
propionate_production.donor
proteolysis.pre
saccharolysis.pre
lipolysis.pre
acetate_production.pre
butyrate_production.pre
mucin_degradation.pre
propionate_production.pre
proteolysis.post
saccharolysis.post
lipolysis.post
acetate_production.post
butyrate_production.post
mucin_degradation.post
propionate_production.post
dist_gmm.donor_pre
dist_gmm.post_pre
motility
sporulation
oxygen.facultative
saccharolysis
proteolysis
lipolysis
mucin_degradation
acetate_production
propionate_production
butyrate_production
present_pre_FMT
abd_ratio.donor_pre
allele_dist.donor_pre
allele_div.donor.norm
allele_div.pre.norm
allele_div.post.norm
NA
cor
0.4
0.0
−0.4

study_id	disease_indication	study_ref	n_patients	n_allogenic_fmts	n_full_triads	n_autologous_fmts	raw_data_accession
MetS_NL_1	metabolic syndrome	Li et al, 2016 (PMID: 27126044); Kootte et al, 2017 (PMID: 28978426); **this study**	26	16	16	10	PRJEB12357,PRJEB46778
UC_NL	ulcerative colitis	Rossen et al, 2015 (PMID: 25836986); **this study**	27	16	13	11	PRJEB46777
ABXR_NL	antibiotics resistance	Singh et al, 2018 (PMID: 25836986); **this study**	14	19	15	0	PRJEB46779
div_AU	recurrent *C. difficile* infection; ulcerative colitis	**this study**	5	5	5	0	PRJEB46780
MetS_NL_Koopen	metabolic syndrome	Koopen et al, 2021 (PMID: 34177842)	24	12	12	12	PRJEB44237
ABXR_div_Leo	antibiotics resistance	Leo et al, 2020 (PMID: 32585945)	26	26	16	0	PRJEB35816
ABXR_IS_BarYoseph	antibiotics resistance	Bar-Yoseph et al, 2021 (PMID: 32511695)	14	14	12	0	PRJNA628604
RCDI_US_Smillie	recurrent *C. difficile* infection	Smillie et al, 2018 (PMID: 29447696)	19	22	19	0	PRJEB23524
RCDI_US_Aggarwala	recurrent *C. difficile* infection	Aggarwala et al, 2021 (PMID: 34580445)	18	14	13	0	PRJNA637878
RCDI_US_Watson	recurrent *C. difficile* infection	Watson et al, 2021 (biorxiv)	10	10	10	0	PRJNA701961
RCDI_US_Podlesny	recurrent *C. difficile* infection	Podlesny et al, 2022 (PMID: 35337386)	8	8	8	0	PRJEB39023
RCDI_US_Moss	recurrent *C. difficile* infection	Moss et al, 2017 (PMID: 28827811)	6	6	2	0	PRJNA349197
UC_US_Damman	ulcerative colitis	Damman et al, 2015 (PMID: 26288277)	6	6	6	0	PRJNA285502
UC_US_Nusbaum	ulcerative colitis	Nusbaum et al, 2018 (PMID: 30010747)	4	4	4	0	PRJNA438164
UC_US_Lee	ulcerative colitis	Lee et al, 2017 (PMID: 28473000)	2	2	2	0	PRJNA353655
CD_US_Vaughn	Crohn's disease	Vaughn et al, 2016 (PMID: 27542133)	18	18	15	0	PRJNA321058
IBS_NO_Goll	irritable bowel syndrome	Goll et al, 2020 (PMID: 32991818)	30	30	21	0	PRJEB36140
MEL_US_Davar	melanoma (anti-PD1 treatment)	Davar et al, 2021 (PMID: 33542131)	26	27	15	0	PRJNA672867
MEL_IS_Baruch	melanoma (anti-PD1 treatment)	Baruch et al, 2021 (PMID: 33303685)	10	10	10	0	PRJNA678737
REN_IT_Ianiro	renal carcinoma	Ianiro et al, 2020 (PMID: 32859933)	10	10	7	0	PRJNA643802
TOU_CN_Zhao	Tourette's syndrome	Zhao et al, 2020 (PMID: 33424650)	5	5	5	0	PRJNA628029
CTR_RU_Goloshchapov	healthy volunteers	Goloshchapov et al, 2019 (PMID: 31888470)	3	3	3	0	PRJNA510036

	species presence				
**scenario**	**D**	**R**	**P**	**determinant variants**	**description**
not observed	N	N	N	%	no marker gene coverage in any sample of the donor-pre-post triplet
species loss	N/Y	Y	N	%	species present pre-FMT, but not post FMT
rejection	Y	N	N	%	species present in donor, but not detectable in recipient post-FMT
persistence	N	Y	Y	R > P	recipient strain populations are retained after FMT
persistence (conspecific)	Y	Y	Y	R > 8*D **&** P < (R+D)	recipient strains remain dominant, >8-fold higher than donor strains
coexistence (conspecific)	Y	Y	Y	R < 8*D **&** D < 8*R **&** P < (R+D)	donor and recipient strain populations coexist, at a ratio of >1:8
colonisation (conspecific)	Y	Y	Y	D > 8*R **&** P < (R+D)	donor strains take over, >8-fold higher than recipient strains post-FMT
colonisation	Y	N	Y	D > P	species not present in recipient pre-FMT, donor strains outweigh novel strains post-FMT
novel strains (conspecific)	N	Y	Y	P ≥ R	recipient strains are replaced by strains that are *not* from the donor (but either environmental or previously undetected in donor or recipient)
	Y	Y	Y	P ≥ (R+D)	
novel strains	N	N	Y	%	species not detectable pre-FMT, from environment or blooming
	Y	N	Y	P > D	
**summarised scenarios**					
resilience of recipient strains					persistence, persistence (conspecific) or coexistence (conspecific)
takeover by donor strains					colonisation or colonisation (conspecific)
turnover of recipient strains					colonisation, colonisation (conspecific), novel strains or novel strains (conspecific)

D: donor baseline

R: recipient pre-FMT baseline

P: recipient post-FMT

Scope	Variable	D	R	P	D:R	R:P	Definition
host: clinical & procedural	minimum sequencing depth in triplet	Y	Y	Y			minimum sequencing depth of donor, pre- and post-FMT samples
	indication (rCDI, UC, CD, MetS, ESBL)		Y				indication in recipient, encoded as binary variable (1, 0) for each indication
	bowel preparation		Y				bowel preparation before FMT; confounded with study/indication
	antibiotics pre-treatment		Y				antibiotics usage during 4 weeks leading up to FMT; fully confounded with indication (antibiotics used by rCDI patients)
	stool sample preparation	Y					stool sample preparation for FMT (fresh = 1; frozen = 0)
	FMT route		Y				FMT administration route (nasogastric or nasoduodenal tube = 1; colonoscopy and/or enema = 0)
	clinical success		Y				indication-specific endpoint at latest recorded timepoint post FMT (yes/no)
microbiome: taxonomic diversity	species richness	Y	Y	Y			rarefied species richness, scaled to unit variance
	richness ratio				Y	Y	ratio of species richness (e.g., D/R, P/R)
	functional redundancy	Y	Y	Y			ratio of functional diversity (rarefied KO richness) and species richness, scaled to unit variance
	community dissimilarity				Y	Y	Bray-Curtis dissimilarity of microbiome community composition
	enterotype (*Prev., Bact., Firm.*)	Y	Y	Y			enterotype, classified based on genus abundances
	matching enterotype				Y	Y	does enterotype match between D-R or R-P
	metabolic pathway distance				Y	Y	Aitchinson distance of Gut Metabolic Module composition
microbiome: metabolic potential	proteolysis potential	Y	Y	Y			total fraction of microbiome-encoded metabolic potential, quantified via Gut Metabolic Modules
	saccharolysis potential	Y	Y	Y			
	lipolysis potential	Y	Y	Y			
	mucin degradation potential	Y	Y	Y			
	propionate production potential	Y	Y	Y			
	butyrogenesis potential	Y	Y	Y			
microbiome: species & strain-level	abundance of other species	Y	Y	Y			rel. abd. of other (non-focal) species in the sample, scaled to unit variance
	engraftment of other species			Y			
	abundance of focal species	Y	Y	Y			rel. abd. of focal species, scaled to unit variance
	focal species abundance ratio				Y	Y	ratio of relative abundance of the focal species in D-R or R-P
	phylogenetic complementarity		Y				minimum phylogenetic distance of focal species to recipient community pre-FMT, scaled to unit variance
	functional complementarity		Y				average representation of focal species' KEGG ortholog repertoire in recipient community pre-FMT
	allele distance				Y	Y	Euclidean distance between intra-specific allele frequencies (i.e., distance between strain populations; see Methods)
	strain population diversity	Y	Y	Y			intra-specific population-wide core genome allele diversity (i.e., strain population diversity in focal sample; see Methods)

D: donor baseline
R: recipient pre-FMT baseline
P: recipient post-FMT
