# Supplementary material for: Drivers and determinants of strain dynamics following fecal microbiota transplantation
Source: Nat Med. 2022 Sep 15;28(9):1902–12. doi: 10.1038/s41591-022-01913-0 (PMC9499871; doi:10.1038/s41591-022-01913-0)
Supplement: Supplementary file 2 — Reporting Summary [file 41591_2022_1913_MOESM2_ESM.pdf]

nature portfolio

Corresponding author(s): Peer Bork

Last updated by author(s): Jun 17, 2022

Reporting Summary

Nature Portfolio wishes to improve the reproducibility of the work that we publish. This form provides structure for consistency and transparency in reporting. For further information on Nature Portfolio policies, see our Editorial Policies and the Editorial Policy Checklist.

Statistics

For all statistical analyses, confirm that the following items are present in the figure legend, table legend, main text, or Methods section.

n/a	Confirmed	
☐	☒	The exact sample size (*n*) for each experimental group/condition, given as a discrete number and unit of measurement
☐	☒	A statement on whether measurements were taken from distinct samples or whether the same sample was measured repeatedly
☐	☒	The statistical test(s) used AND whether they are one- or two-sided<br>*Only common tests should be described solely by name; describe more complex techniques in the Methods section.*
☐	☒	A description of all covariates tested
☐	☒	A description of any assumptions or corrections, such as tests of normality and adjustment for multiple comparisons
☐	☒	A full description of the statistical parameters including central tendency (e.g. means) or other basic estimates (e.g. regression coefficient) AND variation (e.g. standard deviation) or associated estimates of uncertainty (e.g. confidence intervals)
☐	☒	For null hypothesis testing, the test statistic (e.g. *F*, *t*, *r*) with confidence intervals, effect sizes, degrees of freedom and *P* value noted<br>*Give P values as exact values whenever suitable.*
☒	☐	For Bayesian analysis, information on the choice of priors and Markov chain Monte Carlo settings
☒	☐	For hierarchical and complex designs, identification of the appropriate level for tests and full reporting of outcomes
☐	☒	Estimates of effect sizes (e.g. Cohen's *d*, Pearson's *r*), indicating how they were calculated

Our web collection on statistics for biologists contains articles on many of the points above.

Software and code

Policy information about availability of computer code

Data collection	No software was used for data collection (see section 'Data' below).
Data analysis	Metagenomic read processing, profiling, assembly and calling of Single Nucleotide Variants was performed using established pipelines and tools. Data was analysed using the statistical computing framework R (v4.0.4), based on previously published algorithms with some adaptations, as outlined in the Methods. Analysis code is available via github (https://github.com/grp-bork/fmt_metastudy); pre-processed source data via Zenodo (DOI 10.5281/zenodo.6611040).

For manuscripts utilizing custom algorithms or software that are central to the research but not yet described in published literature, software must be made available to editors and reviewers. We strongly encourage code deposition in a community repository (e.g. GitHub). See the Nature Portfolio guidelines for submitting code & software for further information.

Data

Policy information about availability of data

All manuscripts must include a data availability statement. This statement should provide the following information, where applicable:

- Accession codes, unique identifiers, or web links for publicly available datasets
- A description of any restrictions on data availability
- For clinical datasets or third party data, please ensure that the statement adheres to our policy

Raw metagenomic sequencing data have been uploaded to the European Nucleotide Archive under the accession numbers PRJEB46777, PRJEB46778, PRJEB46779 and PRJEB46780. Publicly available datasets used in this study were identified and downloaded manually; the full list, including accession codes and PMIDs, is available as supplementary table. Contextual data for participants was manually curated by several expert curators and is available as online supplementary material. Metagenome-assembled genomes are available for download via Zenodo (DOI 10.5281/zenodo.5534163).

Field-specific reporting

Please select the one below that is the best fit for your research. If you are not sure, read the appropriate sections before making your selection.

☒ Life sciences ☐ Behavioural & social sciences ☐ Ecological, evolutionary & environmental sciences

For a reference copy of the document with all sections, see nature.com/documents/nr-reporting-summary-flat.pdf

Life sciences study design

All studies must disclose on these points even when the disclosure is negative.

Sample size	A literature search for publicly available metagenomic datasets was conducted and all that met quality criteria were included, in addition to the newly generated data first described by our study. No pre-calculations of sample size were conducted. Most of our reported findings rely on LASSO models that were built in 80:20 cross-validation; only species with sufficient observations (≥50 FMTs) were therefore chosen for LASSO modeling.
Data exclusions	Few (≤10) metagenomic samples were excluded prior to analysing the data as they either had suspiciously low sequencing depths after quality filtering or had unclear/conflicting annotated metadata.
Replication	LASSO models were built using cross-validation setups and reported results were averaged over validation folds. Moreover, our entire dataset more than doubled in size during the revision (from 142 FMTs to 316 FMTs studied), but results were qualitatively and quantitatively reproduced on this larger set.
Randomization	No randomization was conducted in the 'div_AU' study due to the small cohort size (n=5). Randomization information for other cohorts used in this study can be found in the respective original publications.
Blinding	Blinding information for cohorts used in this study can be found in the respective original publications. All new metagenomic sequencing was performed by 'blinded' technicians at the European Molecular Biology Laboratory (Heidelberg, Germany).

Reporting for specific materials, systems and methods

We require information from authors about some types of materials, experimental systems and methods used in many studies. Here, indicate whether each material, system or method listed is relevant to your study. If you are not sure if a list item applies to your research, read the appropriate section before selecting a response.

Materials & experimental systems

n/a	Involved in the study	
☒	☐	Antibodies
☒	☐	Eukaryotic cell lines
☒	☐	Palaeontology and archaeology
☒	☐	Animals and other organisms
☐	☒	Human research participants
☐	☒	Clinical data
☒	☐	Dual use research of concern

Methods

n/a	Involved in the study	
☒	☐	ChIP-seq
☒	☐	Flow cytometry
☒	☐	MRI-based neuroimaging

Human research participants

Policy information about studies involving human research participants

Population characteristics	Study population were patients (males and females aged 7-90 years) undergoing a fecal microbiota transplantation procedure for the treatment of: recurrent Clostridioides difficile infection (n=62), infection with extended-spectrum beta-lactamase producing bacteria (n=59), metabolic syndrome (n=50), ulcerative colitis (n=42), anti-PD1 therapy resistance in melanoma patients (n=37), irritable bowel syndrome (n=30), Crohn's disease (n=18), chemotherapy-induced diarrhea in renal carcinoma patients (n=10), Tourette's syndrome (n=5) and healthy volunteers (n=3). Detailed per-subject demographic and clinical information on all participants, to the extent available/curatable from public studies, are reported in the supplementary material.<br><br>Inclusion criteria for 'div_AU' study: (1) males and females aged 18-75 years; (2) > 6 month history of active moderate ulcerative colitis (Mayo score of 4-10) OR diarrhoea (> 3 motions/day) in association with a confirmed diagnosis of Clostridioides difficile infection (toxin positive); (3) never had FMT treatment for any reason.
Recruitment	Participants in the 'div_AU' study were consecutively-enrolled patients who were referred to the Centre for Digestive Diseases (CDD, Australia) for treatment of either toxin-positive Clostridioides difficile infection or ulcerative colitis (Mayo score = 4-10) from November 2014 to July 2015 inclusive, met inclusion criteria for the study and were willing to participate. Participants were required to provide stool samples from home once a week after treatment, for one month. Each sample

had to be delivered in person to the CDD within 24 hours of collection for proper storage, thereby restricting the study population to participants residing within short travel distance to the CDD and could manage the logistics involved in sample collection and delivery. However, this potential bias did not impact on the standard of therapy received. Moreover, limiting the time from sample collection to frozen storage minimized variations in the microbial community of the fecal sample arising from environmental changes, thus improving the accuracy of our findings.

Ethics oversight

Centre for Digestive Diseases Human Research Ethics Committee

Note that full information on the approval of the study protocol must also be provided in the manuscript.

Clinical data

Policy information about clinical studies

All manuscripts should comply with the ICMJE guidelines for publication of clinical research and a completed CONSORT checklist must be included with all submissions.

Clinical trial registration

Australian New Zealand Clinical Trials Registry under ACTRN12614000503628 (Universal Trial number: U1111-1156-5909)

Study protocol

Detailed information is deposited on the ANZCTR website (https://www.anzctr.org.au) under the trial number

Data collection

Participants in the 'div_AU' study were consecutively-enrolled patients who were referred to the Centre for Digestive Diseases (CDD, Australia) for treatment of either toxin-positive Clostridioides difficile infection or ulcerative colitis (Mayo score = 4-10), met inclusion criteria for the study and provided written informed consent.

A total of 3 patients with ulcerative colitis and 2 with Clostridioides difficile infection participated in and completed the study. Fecal samples were collected from November 2014 to July 2015 inclusive. These were sent to the European Molecular Biology Laboratory (Heidelberg, Germany) for metagenomic sequencing.
Further details about the study cohort can be found in the Supplementary Material.

Outcomes

This was a small-scale pilot study to explore microbiome-level outcomes (successful colonisation by donor microbes); standard protocols were used to clinically assess remission for patients (who suffered from rCDI or ulcerative colitis).

Primary outcome: Donor microbiota implantation (defined as 50% similarity to the donor) as measured by high-throughput DNA sequencing of bacteria in stool.
Secondary outcome: Relationship between donor microbiota implantation and clinical improvement as defined by a 3 point or greater improvement in Mayo score for ulcerative colitis patients or eradication of Clostridium difficile infection (CDI) and improvement in bowel frequency to 1-2 stools per day in CDI patients.
